# Pore-forming protein complexes from *Pleurotus* mushrooms kill western corn rootworm and Colorado potato beetle through targeting membrane ceramide phosphoethanolamine

**DOI:** 10.1038/s41598-019-41450-4

**Published:** 2019-03-25

**Authors:** Anastasija Panevska, Vesna Hodnik, Matej Skočaj, Maruša Novak, Špela Modic, Ivana Pavlic, Sara Podržaj, Miki Zarić, Nataša Resnik, Peter Maček, Peter Veranič, Jaka Razinger, Kristina Sepčić

**Affiliations:** 1 Department of Biology, Biotechnical Faculty, University of Ljubljana, Jamnikarjeva 101, 1000 Ljubljana, Slovenia; 2 Agricultural Institute of Slovenia, Hacquetova 17, 1000 Ljubljana, Slovenia; 3 Department of Biotechnology, University of Rijeka, Radmile Matejčić 2, 51000 Rijeka, Croatia; 4 Institute of Cell Biology, Faculty of Medicine, University of Ljubljana, Vrazov trg 2, 1000 Ljubljana, Slovenia

## Abstract

Aegerolysins ostreolysin A (OlyA) and pleurotolysin A (PlyA), and pleurotolysin B (PlyB) with the membrane-attack-complex/perforin domain are proteins from the mushroom genus *Pleurotus*. Upon binding to sphingomyelin/cholesterol-enriched membranes, OlyA and PlyA can recruit PlyB to form multimeric bi-component transmembrane pores. Recently, *Pleurotus* aegerolysins OlyA, PlyA2 and erylysin A (EryA) were demonstrated to preferentially bind to artificial lipid membranes containing 50 mol% ceramide phosphoethanolamine (CPE), the main sphingolipid in invertebrate cell membranes. In this study, we demonstrate that OlyA6, PlyA2 and EryA bind to insect cells and to artificial lipid membranes with physiologically relevant CPE concentrations. Moreover, these aegerolysins permeabilize these membranes when combined with PlyB. These aegerolysin/PlyB complexes show selective toxicity toward western corn rootworm larvae and adults and Colorado potato beetle larvae. These data strongly suggest that these aegerolysin/PlyB complexes recognize CPE as their receptor molecule in the insect midgut. This mode of binding is different from those described for similar aegerolysin-based bacterial complexes, or other *Bacillus thuringiensis* Cry toxins, which have protein receptors. Targeting of *Pleurotus* aegerolysins to CPE and formation of transmembrane pores in concert with PlyB suggest the use of aegerolysin/PlyB complexes as novel biopesticides for the control of western corn rootworm and Colorado potato beetle.

## Introduction

Western corn rootworm (WCR; *Diabrotica v. virgifera* LeConte; Coleoptera, Chrysomelidae) is an important pest on maize in the USA^1,2^ and Europe^3^, and it was reported to cause annual economic losses of over 1 billion dollars in the USA^4,5^. In Europe, the control of WCR by crop rotation^3,6^, biological control options^7,8^, and host-plant native resistance and tolerance^9^ are being evaluated, although these approaches have not achieved widespread success in North America^10^. In addition to crop rotation, chemical control mainly focuses on the larva stages of WCR^6^. In 2003, the Environmental Protection Agency approved the first commercial use of transgenic corn hybrids that express proteinaceous crystal toxins (i.e., Cry toxins) from *Bacillus thuringiensis* (e.g., Bt maize) against WCR larvae^11,12^. This group of toxins comprises approximately 300 proteins that have been divided into 75 subgroups. Cry toxins are species specific, and are toxic due to their binding to species-specific surface proteins in the microvilli of the larva midgut cells (e.g., cadherins, aminopeptidases, alkaline phosphatases)^11,12^. Since the discovery of these toxins and their registration as pesticides in the USA in 1961, *B. thuringiensis* has been the most successful pathogen for the control of WCR, and now commands ~2% of the total insecticide market^13^. However, WCR can continuously develop resistance to these toxins through different mechanisms^1,14–16^.

The Colorado potato beetle (CPB; *Leptinotarsa decemlineata* [Say]; Coleoptera, Chrysomelidae) has been driving the modern insecticide industry since the early days of its spread^17^. Neonicotinoid insecticides and some endotoxins from *B. thuringiensis* subsp. *tenebrionis* are generally used for CPB control. However, as for WCR, CPB can continuously develop resistance against these biopesticides through various mechanisms^18,19^.

The search for alternative biopesticides and approaches is therefore of extreme importance, such as the development of efficient attract-and-kill strategies. These efforts very recently resulted in the discovery of novel proteinaceous toxins that are specific for WCR and some other coleoptera. These have been isolated from different bacterial species to *B. thuringiensis*, and include IPD072a from *Pseudomonas chlororapis*^20^, PiP-47a from *Pseudomonas mosselii*^21^, GNIP1Aa from *Chromobacterium piscinae*^22^, and AflP-1A/AflP-1b from *Alcaligenes faecalis*^23^. The *Pseudomonas*-derived toxins IPD072a and PiP-47a do not match any other protein amino-acid sequences currently in databases, while the N-terminal region of GNIP1Aa from *C. piscinae* was assigned to the membrane-attack-complex/perforin (MACPF) protein superfamily. In contrast to the aforementioned proteins that are toxic toward WCR in their monomeric forms, AflP-1A and AflP-1b from *A. faecalis* act as a bi-component toxic complex, in which the AflP-1A partner belongs to the aegerolysin protein family.

The aegerolysins (Pfam 06355; InterPro IPR009413) currently comprise over 350 small (~15–20 kDa), β-structured proteins that are found in several eukaryotic and bacterial taxa^24–26^. The common prominent feature of these proteins is their interactions with specific membrane lipids and lipid domains. Aegerolysins from the fungal genus *Pleurotus* have been shown to interact with sphingomyelin/cholesterol domains in artificial and biological membranes^27–32^. These aegerolysins, namely ostreolysin A (OlyA) from *P. ostreatus* and pleurotolysin A2 (PlyA2) and erylysin A (EryA) from *P. eryngii*, were recently found to interact even more strongly with lipid vesicles that contained equimolar levels of cholesterol and ceramide phosphoethanolamine (CPE)^33^. CPE is the major sphingolipid in invertebrate cell membranes and is an analog of sphingomyelin, which dominates in vertebrates^33–36^. The dissociation constants of OlyA and PlyA2 binding to equimolar CPE/cholesterol and sphingomyelin/cholesterol lipid vesicles were reported to be 10^−8^ M and >10^−5^ M, respectively^33,37^, which suggests that the interactions of these aegerolysins with CPE-containing membrane systems is at least 1000-fold stronger. This discovery led to the use of *Pleurotus* aegerolysins as useful molecular markers of CPE distribution in insect tissues, and for detection of the bloodstream form of *Trypanosoma brucei*^33^. Furthermore, OlyA, OlyA6, and PlyA were shown to combine with pleurotolysin B (PlyB), a 59-kDa protein partner with a MACPF domain that is produced by *P. ostreatus*^27,30,38^, while EryA acts in concert with another MACPF-protein, erylysin B (EryB), which shares 96% amino acid sequence identity with PlyB^39^. OlyA/PlyB, OlyA6/PlyB and PlyA/PlyB combinations can then act as bi-component pore-forming complexes for artificial and biological cell membranes that contain sphingomyelin/cholesterol domains^27,30,31,38,40^. The membrane-disrupting potential of these aegerolysin/PlyB complexes has, however, not been tested on CPE-containing membranes.

The binary and quaternary cytolytic complexes of bacterial origin where aegerolysin-like proteins are combined with larger, non-aegerolysin protein partner(s) that have been described to date comprise: Cry16Aa/Cry17Aa/Cbm17.1/Cbm17.2 from *Clostridium bifermentas* subsp. *Malaysia*^41^; Cry34Ab1/Cry35Ab1 from *B. thuringiensis*^42,43^; and AflP-1A/AflP-1b from *A. faecalis*^23^. These heteromeric aegerolysin-based cytolytic complexes have been exploited as potent biopesticides for specific pests, whereby Cry16Aa/Cry17Aa/Cbm17.1/Cbm17.2 acts against *Aedes* mosquitoes, and Cry34Ab1/Cry35Ab1 and AflP-1A/AflP-1b act against coleoptera species, and mainly WCR. Of the numerous Cry toxins produced by *B. thuringiensis*, Cry34Ab1 is the only aegerolysin-like protein^11^. Cry34Ab1/Cry35Ab1 were genetically introduced into corn hybrids in 2005 (i.e., Bt maize) as tools for the control of WCR larvae^44,45^. Although no specific Cry34Ab1/Cry35Ab1 receptors have been identified to date, the data obtained so far suggest that they interact with (glyco)protein receptors in the membranes of epithelial cells in the insect midgut, with the resultant damage due to their perforation of these cells^46,47^. WCR larvae were recently reported to evolve resistance to the Bt maize that produces Cry34Ab1/Cry35Ab1^48^. Recently, it was also shown that laboratory-generated WCR colonies selected for resistance to Cry34Ab1/Cra35Ab1 can feed on AflP-1A/AflP-1b–transfected maize, indicating the similar mode of action of these two protein complexes^23^, and urging further searches for alternative biopesticides.

Due to their specific interactions with the insect-specific sphingolipid CPE, it can be assumed that *Pleurotus* aegerolysins OlyA, PlyA2, and EryA can target this lipid in cell membranes of insect pests and form pores in the presence of their MACPF-protein partners. The aim of this study was therefore to determine the toxic potential of these aegerolysin/PlyB complexes on major insect pests such as the Coleoptera species WCR (*Diabrotica virgifera virgifera)*, CPB (*Leptinotarsa decemlineata*) and mealworm (*Tenebrio molitor*), as well as the grain aphid (*Sitobion avenae*, Hemiptera), the greater wax moth (*Galleria mellonella*, Lepidoptera), and the spotted wing *Drosophila* (*Drosophila suzukii*, Diptera). Furthermore, to elucidate the molecular mechanisms of their toxicities, the binding of *Pleurotus* aegerolysins to biological and artificial membranes that contain biologically relevant CPE concentrations, and the permeabilization of these membranes by the aegerolysin/PlyB complexes, were studied in detail. Since EryA was not applied in combination with its endogenous MACPF-protein partner, EryB, the results obtained with EryA/PlyB complexes were not quantitatively compared with those obtained with OlyA6/PlyB and PlyA2/PlyB combinations.

## Results

### *Pleurotus* aegerolysins and aegerolysin/PlyB complexes interact with artificial and biological lipid membranes that contain ceramide phosphoethanolamine

#### Binding to membranes

**Figure 1:**
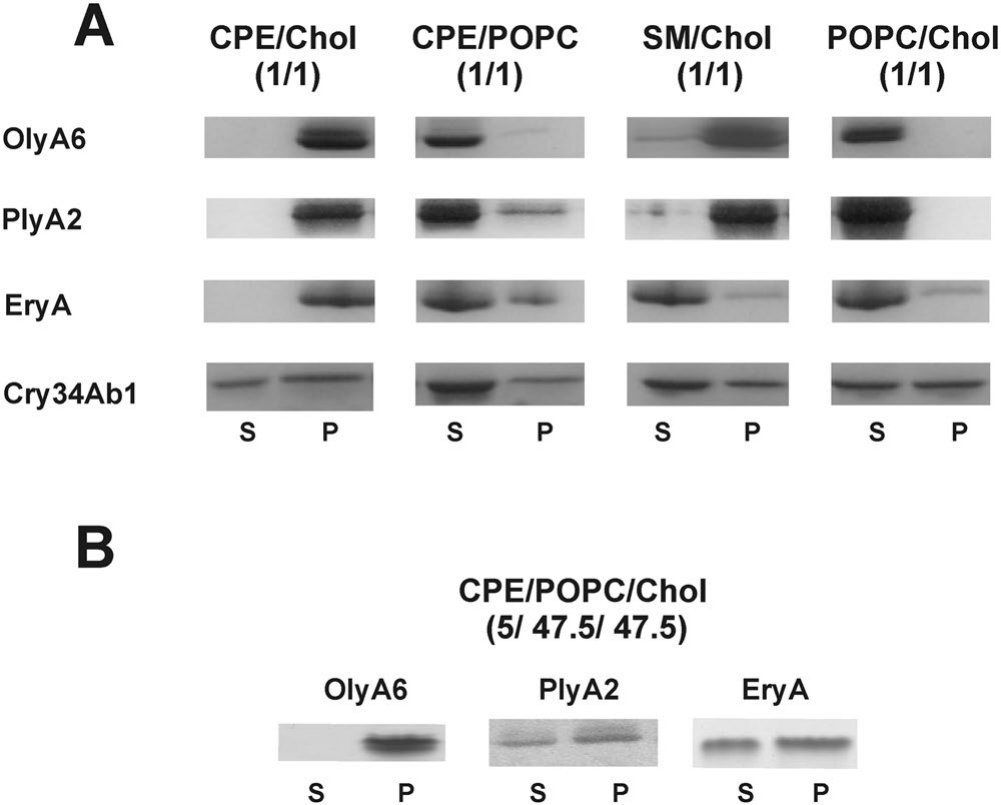
Specificity of aegerolysins for binding to multilamellar lipid vesicles with various lipid compositions. The aegerolysins were incubated with multilamellar vesicles and centrifuged as described in the Methods. The supernatant (S) and pellet (P) fractions were subjected to SDS-PAGE and stained with SimplyBlue SafeStain. (**A**) Binding of OlyA6, PlyA2, EryA, and Cry34Ab1 to vesicles composed of equimolar lipid mixtures, as indicated. (**B**) Binding of OlyA6, PlyA2, and EryA to vesicles composed of CPE, POPC, and cholesterol (molar ratio, 5/47.5/47.5). Control experiments without the vesicles showed no sedimentation of the aegerolysins. CPE, ceramide phosphoethanolamine; Chol, cholesterol; SM, sphingomyelin; POPC, 1-palmitoyl-2-oleoyl-*sn*-glycero-3-phosphocholine.

Using sedimentation assays, we initially confirmed previously reported data by Bhat *et al*.^33^ that OlyA6, PlyA2, and EryA bind to equimolar CPE/cholesterol multilamellar vesicles, while no interactions, or negligible binding, was observed with CPE/1-palmitoyl-2-oleoyl-*sn*-glycero-3-phosphocholine (POPC) and POPC/cholesterol vesicles (Fig. 1A, Supplementary Fig. S1). We also confirmed that while OlyA6 and PlyA2 bind to sphingomyelin/cholesterol multilamellar vesicles, EryA was recovered in the supernatant after centrifugation of these vesicles, which suggested no association of EryA with these lipid membranes^33^. Cry34Ab1 is an aegerolysin-like protein from *B. thuringiensis*, and it bound nonspecifically to all of the forms of the multilamellar lipid vesicles.

In invertebrate membranes, however, CPE levels are considerably lower, as they represent around 2 to 6 mol% of the total membrane lipids^49,50^. Here, the *Pleurotus* aegerolysins, and especially OlyA6, bound strongly to the artificial lipid membranes composed of equimolar POPC and cholesterol supplemented with biologically relevant levels (i.e., 5 mol%) of CPE (Fig. 1B, Supplementary Figs S2 and S3). As shown for OlyA6 and EryA, these two aegerolysins differed most for their binding affinities and lipid specificities; their binding was retained even with CPE levels as low as 1 mol%, which was also dependent on the presence of membrane cholesterol (Supplementary Figs S2–S4). In vesicles containing 5 mol% CPE, OlyA6 was partially recovered in the sediments of vesicles containing 30 mol% and higher cholesterol levels, while EryA binding required higher molar proportions of cholesterol (>30 mol%).

**Figure 2:**
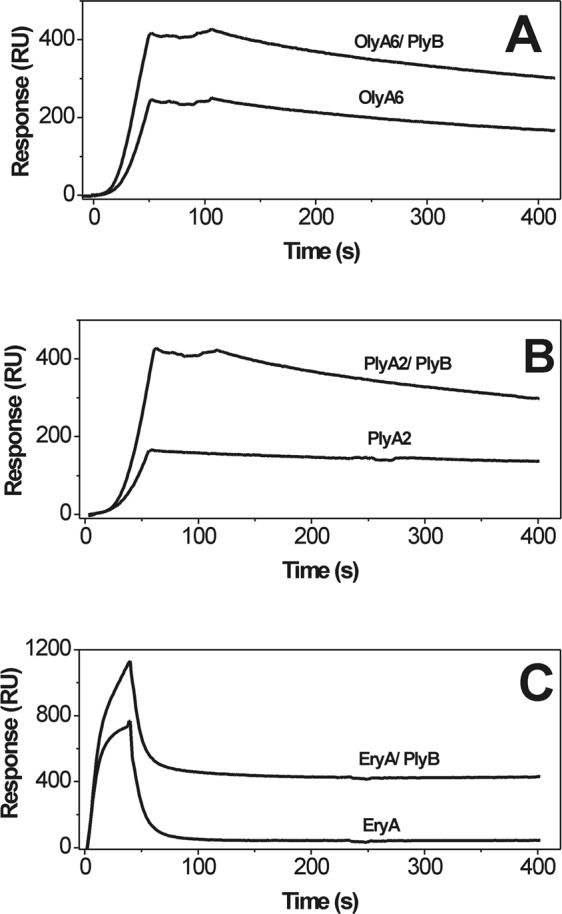
Surface plasmon resonance of the interactions of OlyA6, PlyA2, and EryA with large unilamellar lipid vesicles composed of CPE, POPC, and cholesterol (molar ratio, 5/47.5/47.5). The vesicles were immobilized (Biacore L1 chip) to approximately 7000 RU and the analytes were injected in running buffer (flow rate, 10 μL/min). Representative sensorgrams of triplicate analyses are shown. (**A**–**C**) Binding to lipid vesicles of 0.25 μM OlyA6 alone or in combination with PlyB (**A**), 0.25 μM PlyA2 alone or in combination with PlyB (**B**), and 5 μM EryA alone or in combination with PlyB (**C**) (aegerolysin/PlyB molar ratio, 12.5/1).

Surface plasmon resonance studies with on-chip immobilized large unilamellar vesicles (LUVs; Fig. 2) confirmed the data obtained in the sedimentation assays, and provided deeper insight into the kinetics of the *Pleurotus* aegerolysin interactions with CPE-containing membranes. All of these aegerolysins interacted with LUVs that contained 5 mol% CPE; however, EryA showed the same levels of binding at higher concentrations than those seen for OlyA6 and PlyA2, indicating its weaker interaction with these LUVs. Also, in contrast to OlyA6 and PlyA2, the binding kinetics of EryA during the dissociation phase indicated its reversible interaction with the lipid membranes. In line with the sedimentation assay, the surface plasmon resonance confirmed that the binding of both OlyA6 and EryA to LUVs increased with increased CPE content, and that this was cholesterol dependent (Supplementary Figs S5, S6). Finally, the interactions of all three aegerolysins with the LUVs, including EryA that was not used in combination with its endogenous protein partner (EryB), were considerably stronger in the presence of PlyB (Fig. 2), which suggested the formation of more stable proteolipid complexes.

**Figure 3:**
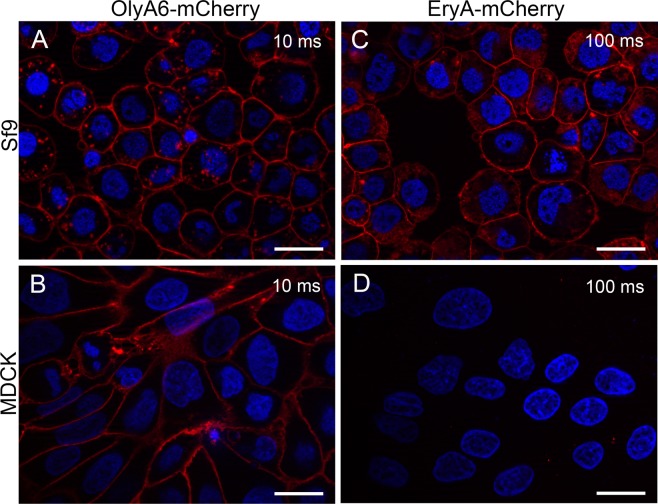
Representative fluorescence microscopy images of cell-surface labeling of insect and mammalian cells with OlyA6 and EryA. Sf9 and MDCK cells were labeled with 5 µM OlyA6-mCherry or 5 µM EryA-mCherry, as described in the Methods. Numbers on images indicate fluorescence acquisition times. Scale bars, 20 µm.

In agreement with the artificial lipid systems used, the binding of the fluorescent derivatives of OlyA6 and EryA was assayed on the insect Sf9 cells (which contain CPE) and the mammalian MDCK cells (which contain sphingomyelin). These data confirmed the binding of OlyA6-mCherry to both cell types (Fig. 3A,B), while EryA-mCherry bound only to the insect Sf9 cells (Fig. 3C,D). Preincubation of OlyA6-mCherry with equimolar CPE/POPC/cholesterol multilamellar vesicles abolished the binding to MDCK cells, whereas preincubation with the same amount of equimolar SM/cholesterol multilamellar vesicles did not significantly affect this binding (Supplementary Fig. 7), again indicating the preference of the aegerolysin protein for CPE over SM.

#### Membrane permeabilization

**Figure 4:**
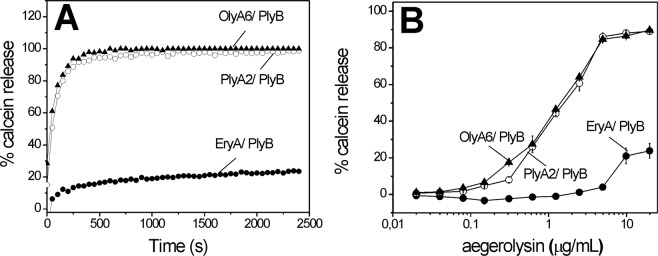
Permeabilization of small unilamellar vesicles composed of CPE, POPC, and cholesterol (molar ratio, 5/47.5/47.5) by OlyA6/PlyB, PlyA2/PlyB, and EryA/PlyB. Fluorescence intensity of calcein released from the lipid vesicles, monitored as described in the Methods. (**A**) Permeabilization of lipid vesicles by 10 μg/mL aegerolysin with PlyB (molar ratio, 12.5/1). (**B**) Concentration dependence of lipid vesicle permeabilization for OlyA6/PlyB, PlyA2/PlyB, and EryA/PlyB. Data are means ± standard error from three repetitions.

By monitoring the fluorescence of calcein released from the small unilamellar lipid vesicles that contained 5 mol% CPE, the concentration-dependent membrane permeabilization by OlyA6, PlyA2, and EryA when combined with PlyB was confirmed (aegerolysin/PlyB molar ratio, 12.5/1) (Fig. 4). The aegerolysins alone or PlyB alone did not have any membrane-disrupting activities (data not shown). The membrane disruption by OlyA6/PlyB and PlyA2/PlyB was comparable, and in line with the membrane binding assays, this was considerably stronger than for EryA/PlyB (Fig. 4A,B). Further analysis of the membrane permeabilization by OlyA6/PlyB using vesicles composed of different equimolar lipid mixtures showed the highest permeabilization of CPE/cholesterol membranes (Supplementary Fig. S8A). OlyA6/PlyB also permeabilized CPE/POPC vesicles, which suggested that the cholesterol content is a facilitating, rather than necessary, factor for the membrane-permeabilizing activity of OlyA6/PlyB complexes (Supplementary Fig. S8A). No permeabilization of POPC/cholesterol vesicles was observed, while the sphingomyelin/cholesterol vesicles permeabilization by OlyA6/PlyB was ~10-fold lower than the permeabilization of CPE/cholesterol membranes. In line with the membrane binding studies, the membrane-permeabilizing activity of OlyA6/PlyB increased with increasing CPE (Supplementary Fig. S8B) and cholesterol (Supplementary Fig. S8C) membrane content.

**Figure 5:**
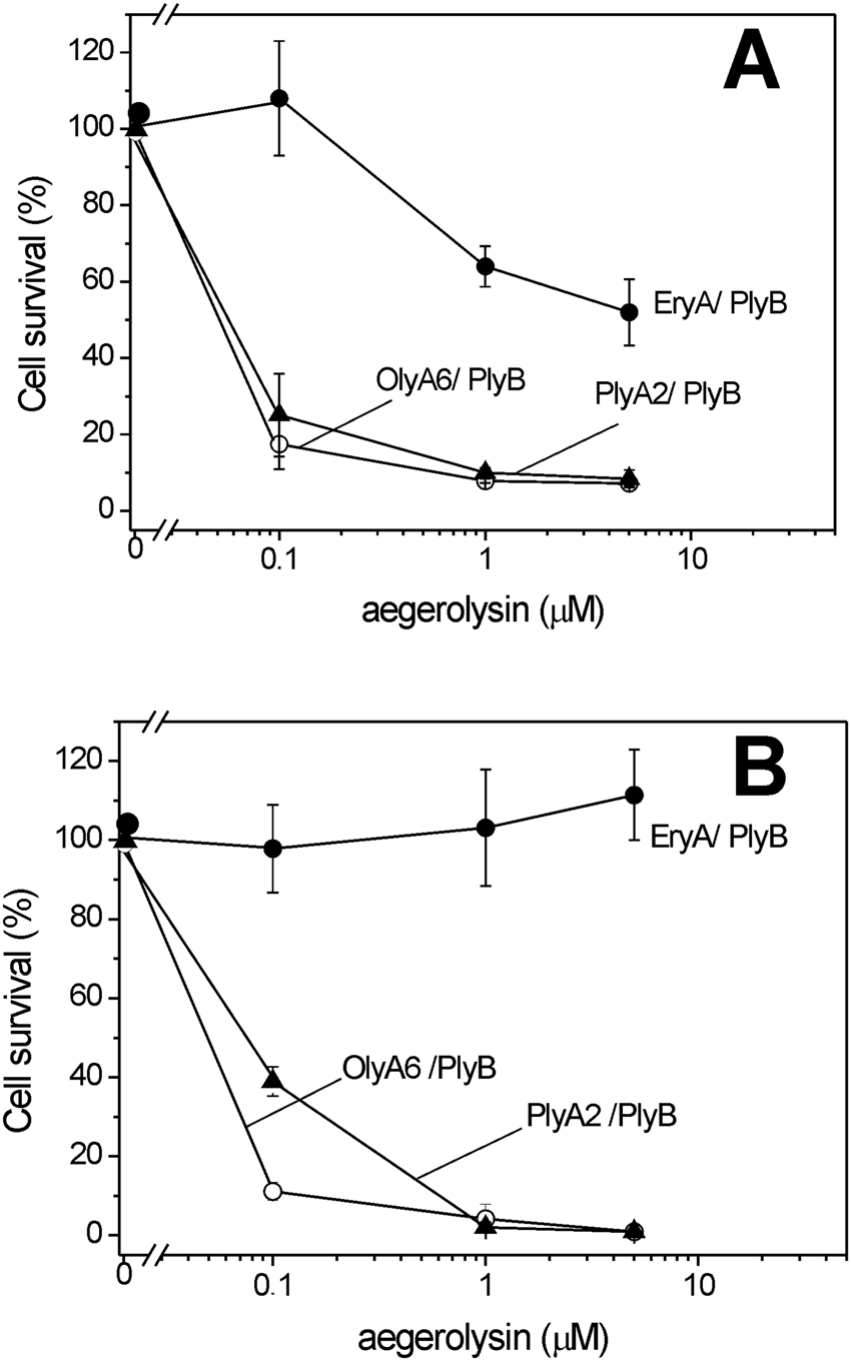
Effects of OlyA6/PlyB, PlyA2/PlyB, and EryA/PlyB on insect Sf9 cell and mammalian MDCK cell survival. Survival rates of Sf9 (**A**) and MDCK (**B**) cells treated with OlyA6/PlyB, PlyA2/PlyB, and EryA/PlyB (as indicated), expressed as the ratio between the luminescence of the treated and the control cells (x100) after 30 min of exposure. Data are means ± SD from three independent experiments.

According to the cell viability assays, the *Pleurotus* aegerolysins OlyA6, PlyA2, and EryA alone (final concentration, 5 µM) were not toxic to the Sf9 and MDCK cells after 30 min of incubation (data not shown). However, when combined with PlyB in the aegerolysin/PlyB molar ratio of 12.5/1, OlyA6 and PlyA2 lysed both of these tested cell lines at all of their tested concentrations, and to similar extents (Fig. 5). In line with the permeabilization using the CPE-enriched artificial lipid vesicles (Fig. 4), EryA/PlyB was a little less toxic to the Sf9 cells than OlyA6/PlyB and PlyA2/PlyB (Fig. 5A). Furthermore, EryA/PlyB did not permeabilize the MDCK cells (Fig. 5B), which is in line with their lack of binding to mammalian cells that contain sphingomyelin instead of CPE (Figs 1A and 3D**)**^33^.

### *Pleurotus* aegerolysin/PlyB complexes are selectively toxic to Western corn rootworm and Colorado potato beetle

The data from the exposure of the various insect pests to OlyA6/PlyB, PlyA2/PlyB, and EryA/PlyB indicated their selective affinity against WCR and CPB, with no effects seen on the survival and behaviour of *T. molitor, D. suzukii, S. avenae*, and *G. melonella* (Supplementary File S1, Fig. S9, Table 1).

**Table 1:** Insecticidal activities of the *Pleurotus* aegerolysin/PlyB complexes against the various insects.

Order	Species	Common name	Aegerolysins (μg/mL)	Effects
Coleoptera	*Tenebrio molitor*	Mealworm	500	Inactive
	*Diabrotica virgifera virgifera*	Western corn rootworm (adults)	500	Death
	*Diabrotica virgifera virgifera*	Western corn rootworm (larvae)	5.6*	Death
	*Leptinotarsa decemlineata*	Colorado potato beetle (larvae)	9.0*	Death
Lepidoptera	*Galleria mellonella*	Greater wax moth (caterpillars)	500	Inactive
Hemiptera	*Sitobion avenae*	Grain aphid	500	Inactive
Diptera	*Drosophila suzukii*	Spotted wing drosophila	500	Inactive

*μg/cm^2^.

**Figure 6:**
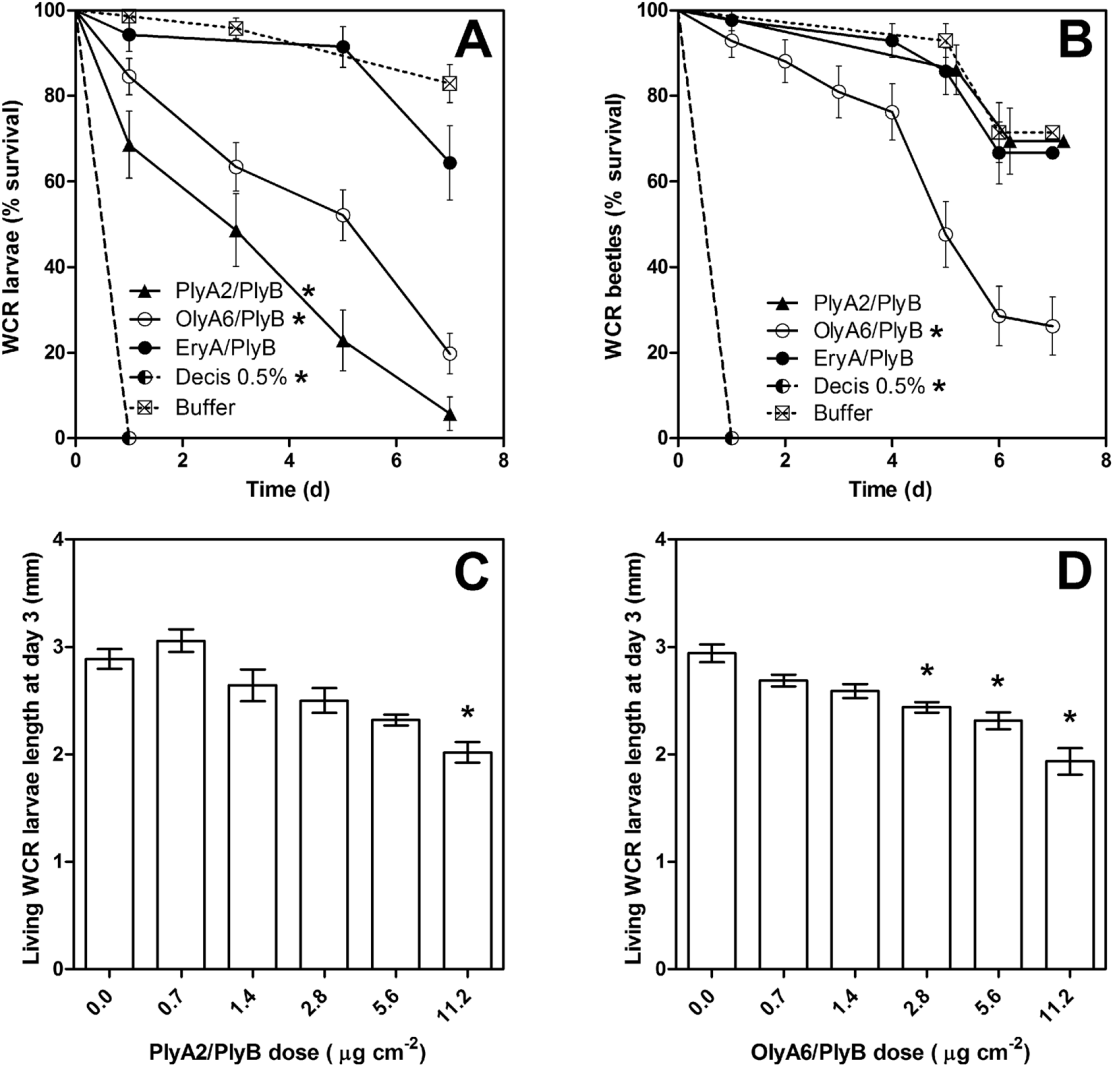
Survival of western corn rootworm larvae and beetles and length of the living larvae following exposure to aegerolysin/PlyB complexes and to insecticide (active ingredient, deltamethrin; Decis). Kaplan-Meier survival curves for the larvae (**A**) and beetles (**B**). Effects of different concentrations of PlyA/PlyB (**C**) and OlyA6/PlyB (**D**) on the length of the living larvae on day 3. *P < 0.05, between treatments and negative control buffer. (**B**) Data points shifted by +0.2 units to prevent overlapping.

Exposure of the WCR larvae to the artificial diet that contained aegerolysin/PlyB or the insecticide (Decis) had significant effects on their survival over these 7-day bioassays (χ^2^ = 120.8; P < 0.0001), whereby PlyA2/PlyB, OlyA6/PlyB, and Decis significantly increased the larva mortality (Fig. 6A, Table 2). Various concentrations of the aegerolysin/PlyB complexes had significant detrimental effects on the WCR larvae when evaluated on day 3 (PlyA2/PlyB, P = 0.002; OlyA6/PlyB, P < 0.0001). PlyA2/PlyB caused significant reduction of the larva length at 11.2 µg/cm^2^, and OlyA6/PlyB at 2.8, 5.6 and 11.2 µg/cm^2^ (Fig. 6C,D).

**Table 2:** Estimates of LT_50_ and LD_50_ of the *Pleurotus* aegerolysin/PlyB protein complexes against the WCR larvae and beetles and the CPB larvae.

Sample	Treatment	LT_50_ (days)^a^		Hazard ratio^b^	χ^2,c^	P-value^c^	LD_50_ (µg/cm^2^)^d^	
		Mean ± SE	95% CI				Mean ± SE	95% CI
WCR larvae	OlyA6/PlyB	4.1 ± 0.4	3.4–4.9	8.4	60.4	<0.0001	7.8 ± 3.6	0.21–15.4
	PlyA2/PlyB	2.3 ± 0.3	1.7–2.8	37.2	84.1	<0.0001	7.4 ± 3.4	0.24–14.5
	EryA/PlyB	7.4 ± 0.3	6.7–8.1	2.7	4.1	>0.05		
	0.5% Decis^e^	0.6 ± 0.001	ND	3670	70.8	<0.0001		
	Buffer	12 ± 2.9 d	5.7–18					
WCR beetles	OlyA6/PlyB	5.0 ± 0.58	3.7–6.2	5.0	22.0	<0.0001		
	PlyA2/PlyB	8.3 ± 1.6	4.9–12	1.2	0.1	>0.05		
	EryA/PlyB	8.9 ± 1.9	4.7 13	1.3	0.4	>0.05		
	0.5% Decis^e^	~0.38	ND	70.5	65.0	<0.0001		
	Buffer	8.4 ± 1.1	6.0–11					
CPB larvae L1 + L2	OlyA6/PlyB	4.3 ± 0.5	3.3–5.4	8.5	22.4	<0.0001		
	PlyA2/PlyB	4.6 ± 0.6	3.3–6.0	6.7	15.9	<0.0001		
	EryA/PlyB	4.9 ± 0.4	3.9–5.9	7.3	14.0	0.0002		
	0.1% Actara^f^	~0.8	ND	111	98.3	<0.0001		
	Buffer	6.7 ± 1.1	4.5–9.0					
CPB larvae L3 + L4	OlyA6/PlyB	4.5 ± 0.3	3.9–5.1	8.1	20.8	<0.0001		
	PlyA2/PlyB	4.1 ± 0.3	3.5–4.7	12.3	32.6	<0.0001		
	EryA/PlyB	5.4 ± 0.4	4.6–6.2	4.0	6.1	>0.05		
	0.1% Actara^f^	~0.9	ND	129	106	<0.0001		
	Buffer	9.6 ± 1.8	2.9–31.6					

^a^WCR larvae were exposed to 5.6 µg/cm^2^ aegerolysin/PlyB. WCR beetles were exposed to 0.5 mg/mL aegerolysin and 0.04 mg/mL PlyB. CPB larvae were exposed to 9.0 µg/cm^2^ aegerolysin/PlyB. ^b^Hazard ratio is defined as the slope of the survival curve, as a measure of how rapidly the subjects died. The hazard ratio was computed for each treatment from the survival curves, compared to the buffer (negative control). ^c^χ^2^ (log-rank test) was computed separately for each pairwise comparison of treatment versus buffer (negative control), and consequently its corresponding P-value compared to Bonferroni-corrected significance thresholds (P/K, where P = 0.05 and K is the number of pairwise comparisons). If the P-value exceeded the Bonferroni-corrected significance threshold, it is reported as >0.05. ^d^LD_50_ of OlyA6/PlyB and PlyA2/PlyB assessed only for WCR larvae at day 7. ^e^Insecticide based on active ingredient: deltamethrin. ^f^Insecticide based on active ingredient: thiametoxam. ND, not determined.

Exposure of the WCR beetles to the artificial diet containing the aegerolysin/PlyB complexes or the insecticide (Decis) also had significant effects on their survival over these 7-day bioassays (χ^2^ = 139.3; P < 0.0001), whereby OlyA6/PlyB and the insecticide significantly increased beetle mortality (Fig. 6B, Table 2). The individual aegerolysins, PlyB alone, and buffer did not significantly affect the WCR larvae survival or length, or the WCR beetle survival (data not shown).

**Figure 7:**
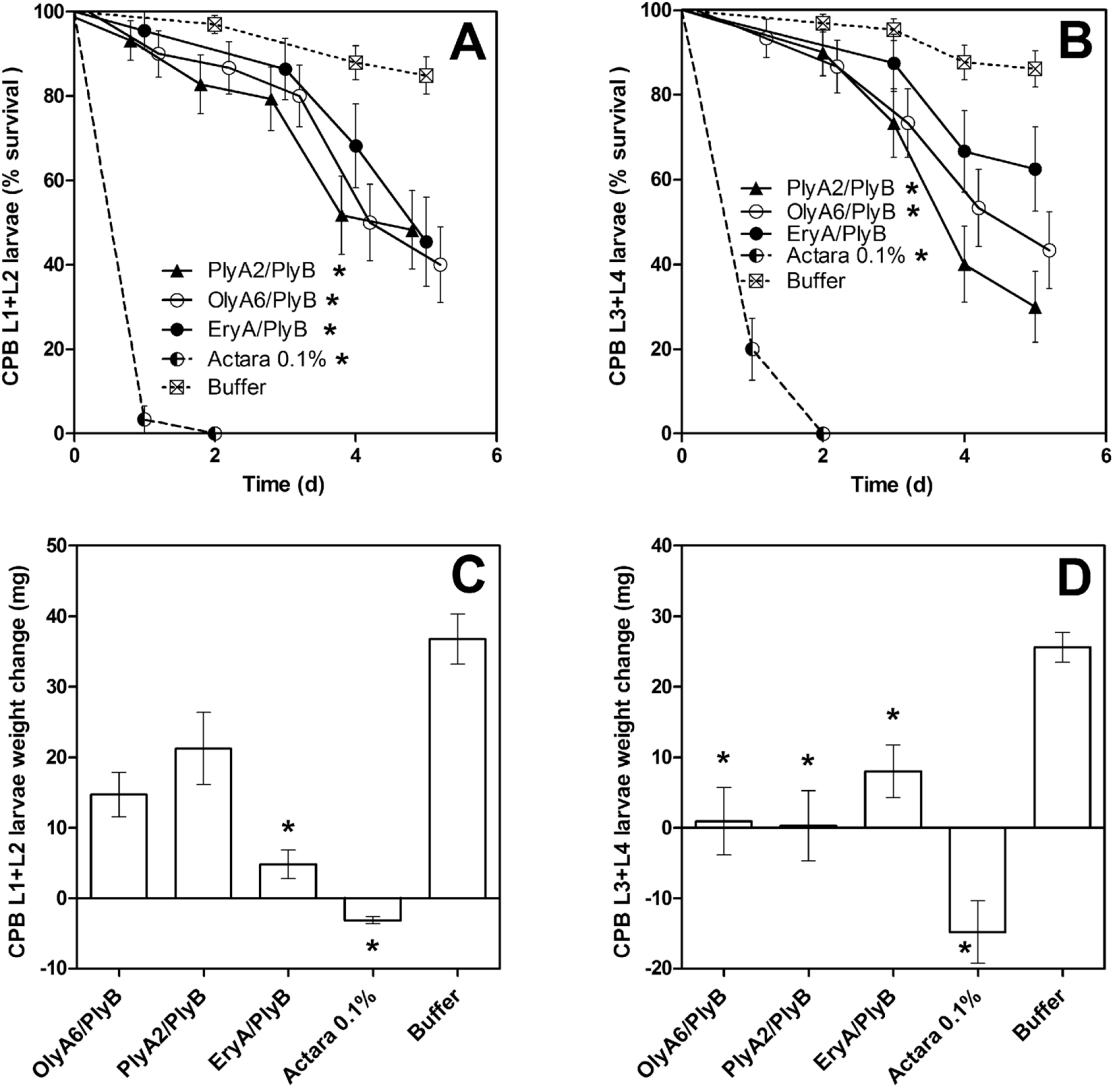
Survival and weight changes of Colorado potato beetle larvae following exposure to aegerolysin/PlyB complexes and to insecticide (active ingredient, thiamethoxam; Actara). Kaplan-Meier survival curves for the L1 + L2 (**A**) and L3 + L4 (**B**) larvae. Weight changes of the L1 + L2 (**C**) and L3 + L4 (**D**) larvae over the 5-day bioassay. *P < 0.05, between treatments and negative control buffer. (**A**,**B**) Data points shifted ±0.2 units to prevent overlapping.

Exposure of the CPB larvae to the leaf discs that were treated with the aegerolysin/PlyB complexes (9.0 µg/cm^2^) or the insecticide (0.1% Actara) had significant effects on their survival (χ^2^ = 136.7; P < 0.0001) and weight changes over these 5-day bioassays. The aegerolysin/PlyB complexes and the insecticide significantly increased larva mortality, with the exception of EryA/PlyB with the L3 + L4 CPB larvae (Fig. 7A,B, Table 2). It should be stressed however that the insecticidal effects of EryA/PlyB complexes cannot be directly compared to those obtained with OlyA6/PlyB and PlyA2/PlyB combinations, since EryA was not used in combination with its endogenous protein partner, EryB. The aegerolysin/PlyB complexes and the insecticide also had significant effects on the CPB larva feeding, as indicated by the changes in the larva weights (L1 + L2 and L3 + L4, P < 0.0001). Further, all of the aegerolysin/PlyB complexes and the insecticide significantly reduced the larva weight gain for L3 + L4 (Fig. 7C,D). The individual aegerolysins, PlyB alone, and the buffer did not significantly affect the L1 + L2 or L3 + L4 larva survival or weight changes (data not shown).

## Discussion

The *Pleurotus* aegerolysin proteins OlyA6, PlyA2, and EryA target the invertebrate membrane sphingolipid CPE and recruit the MACPF protein, PlyB, to consequently form transmembrane lytic complexes. Here, we have shown these interactions might be useful in the further development of novel insect biopesticides. Such specificity, in turn, renders these aegerolysin/PlyB complexes less toxic to taxa outside their primary specificity range, which is an important requirement in pest control management^51^.

Previous studies have described the interactions of OlyA6, OlyA, PlyA, PlyA2, and EryA with lipid vesicles composed of equimolar proportions of sphingomyelin/cholesterol or CPE/cholesterol in detail^28,30,32,33,52^. The potential of the aegerolysins to be used as biomarkers of membrane rafts and for defining the CPE distribution in invertebrates have also been reported^31–33,37,53^. Here, however, we have initially demonstrated that these aegerolysins can bind efficiently to artificial lipid vesicles with particularly low CPE concentrations compared to those reported previously. Our data demonstrate that the aegerolysin/PlyB complexes are formed even at CPE concentrations below 5 mol%, which is comparable to the concentrations in insect cell membranes^50^. OlyA6 and PlyA2 show comparable affinities and are more potent than EryA toward CPE-containing vesicles, and they can also bind to sphingomyelin/cholesterol membranes. EryA, in contrast, recognized exclusively the CPE/cholesterol lipid mixtures, as reported previously^33^. The loss of the choline interaction of EryA was suggested to be dependent on the presence of a cation-π box in PlyA2 (W28/Y91) and OlyA (W28/S91) only, which facilitates the interaction of these two aegerolysins with the choline headgroup of sphingomyelin^33^. Our investigations here with insect and mammalian cell cultures further support the specific interactions of the fluorescently labeled *Pleurotus* aegerolysins with the invertebrate-specific lipid acceptor. Thus, while EryA-mCherry interacted with the insect cells, it showed a lack of binding to the mammalian cells. Indeed, there has been recent confirmation of the presence of several CPE species in lipid extracts of Sf9 cells (Graziano Guella, University of Trento, Italy; personal communication).

In contrast to the *Pleurotus* aegerolysins, bacterial Cry34Ab1 did not show any specific interactions with the lipid mixtures tested in our sedimentation assays here. This is in agreement with the identification of several candidate protein receptors for Cry34Ab1 in brush-border vesicles prepared from the WCR midgut^47^. Similarly, insect membrane proteins have been suggested to be the receptors for the recently discovered aegerolysin-like AflP-1A protein from *A. faecalis*^23^.

It has been reported that the combination of OlyA or PlyA with PlyB readily forms 13-meric transmembrane pores in natural and artificial membranes that contain sphingomyelin/cholesterol domains^28,30,38,54^. However, our data in the present study obtained with artificial lipid vesicles and with insect cell cultures also demonstrate the permeabilization activities of the OlyA6/PlyB, PlyA2/PlyB, and EryA/PlyB complexes toward CPE-containing membranes. Importantly, this membrane permeabilization can be observed at <5 mol% CPE; i.e., at biologically relevant CPE concentrations. The lower membrane-disrupting potential of EryA/PlyB seen here might be explained by the observed lower affinity of EryA toward CPE/cholesterol membranes, and also because EryA was not used in combination with its endogenous pore forming partner protein EryB^39^, which however shares high amino acid sequence identity with PlyB (96%).

Although both Cry34Ab1^55^ and OlyA^56^ alone have been reported to cause remodeling of biological membranes, in the present study, the individual aegerolysins used and PlyB alone did not have any insecticidal effects on WCR and CPB. Only the combination of these *Pleurotus* aegerolysins and PlyB significantly increased insect mortality and the nonlethal effects, such as reduced larva growth (for WCR) and decreased larva weight (for CPB).

These data on the insecticidal effects on WCR larvae are directly comparable to some previously published data. Shlotter and Storer^57^ reported a LD_50_ of 11 µg/cm^2^ for WCR larvae treated with Cry34Ab1/Cry35Ab1 complexes, which is in the same range as seen here for these *Pleurotus* aegerolysin/PlyB complexes. Shlotter and Storer^56^ also reported that 35 µg/cm^2^ Cry34Ab1/Cry35Ab1 complex was apparently not high enough to kill 50% of CPB. In the present study, the aegerolysin/PlyB complexes were more potent, as 9.0 µg/cm^2^ killed 50% of CPB larvae in 4.1 to 5.4 days.

It is more difficult to directly compare other studies to the present study due to methodological differences. Moellenbeck *et al*.^42^ reported a LC_50_ of 55 µg (cell biomass)/cm^2^ after 4 days of exposure of WCR larvae to a crude lysate of *B. thuringiensis* strain PS149B1, which produced the Cry34Ab1/Cry35Ab1 toxins. If we approximate the aegerolysin input concentrations in the bioassays of the present study in µg/mL, as would be obtained if they were uniformly incorporated within the assay medium, we would obtain a LD_50_ of 33.8 ± 8.5 µg/mL for PlyA/PlyB and 47.0 ± 11.0 µg/mL for OlyA6/PlyB after 5 days of exposure. This is comparable to the findings of Yalpani *et al*.^23^, who reported a LD_50_ for WCR larvae of 30.4 µg/mL after 3 to 4 days of exposure to AflP-1A/AflP-1B from *Alcaligenes faecalis*. The present data for day 3 (PlyA/PlyB LD_50_, 54.9 ± 23.2 µg/mL; OlyA6/PlyB LD_50_, 58.3 ± 11.7 µg/mL) are similar to the findings of Wei *et al*.^21^, who reported a LD_50_ for WCR larvae of 52.5 µg/mL after 4 days of exposure to the insecticidal protein PIP-47Aa from *P. mosselii*. Schellenberger *et al*.^20^ reported a LC_50_ of ~120 µg/mL for IPD072Aa from *Pseudomonas chlororaphis* after 8 days of exposure. Here, we report lower LD_50_ for 7 days of exposure: PlyA/PlyB, 27.4 ± 12.5 µg/mL; OlyA6/PlyB, 29.0 ± 13.2 µg/mL.

The specificity of these *Pleurotus* aegerolysin/PlyB complexes toward WCR and CPB might be due to physiological differences between the species tested. In contrast to the other insects tested, the midgut pH of these Coleoptera species has been reported to be around 6^58-60^, which is also the optimal pH for OlyA6 binding to membrane lipids^61^. Furthermore, aegerolysin-based cytolytic protein complexes might be degraded by proteolytic enzymes present in the midgut of resistant insect species^62,63^, or they might simply not reach their molecular targets due to the presence of glycocalyx in midgut epithelial cells^64^. Finally, in addition to CPE, the presence of an additional (glyco)lipid or (glyco)protein aegerolysin receptor site(s) on insect cell membranes cannot be excluded at present.

The loss of larva weight and decrease in their growth, and their death caused by the aegerolysin/PlyB complexes here are similar to the described effects of *B. thuringiensis* Cry34Ab1/Cry35Ab1 toxins. When these *B. thuringiensis* toxins were ingested, they affected the apical surface of the WCR midgut epithelium. Gross changes in the midgut integrity resulted in starvation and dehydration of the larvae^42,45,55^. Although the receptors for the Cry34Ab1/Cry35Ab1 toxins and aegerolysin/PlyB complexes are obviously different, we would suggest that the pathophysiological effects of these proteins are similar, and are basically accounted for by permeabilization of the insect midgut epithelium cells.

We can conclude that these CPE-binding aegerolysins from fungal genus *Pleurotus* in complexes with the MACPF-like protein partner, PlyB, are selectively toxic to WCR and CPB larvae, and can be used to develop a novel generation of biopesticides produced by genetically modified plants for the control of these pests. Importantly, due to their interactions with their specific insect membrane lipid receptor, and not with pest proteins that can be prone to mutation, the chances of evolving resistance to these aegerolysin proteins should be extremely small. Finally, the bioinsecticidal effects of protein complexes based on *Pleurotus*-derived aegerolysins and MACPF partners suggest that in their natural environment, these complexes might be involved in the defence of oyster mushrooms against insect larvae.

## Materials and Methods

### Materials

#### Chemicals

All chemicals used in the present study were from Sigma–Aldrich (USA) unless specified otherwise. Porcine brain sphingomyelin, wool grease cholesterol, and POPC were from Avanti Polar Lipids (USA), and CPE was from Matreya (USA). These lipids were stored at −20 °C and dissolved in chloroform prior to use. CPE was dissolved in 1 mL chloroform/methanol (9/1, v/v) with the addition of 5 μL Milli-Q water.

#### Cells

Insect cells derived from the ovarian epithelial cells of the fall army worm (*Spodoptera frugiperda*; Sf9 cells; Thermo Fischer Scientific, USA) were maintained in continuous suspension culture under serum-free conditions at 28 °C in Insect XPRESS protein-free insect cell medium with L-glutamine (Lonza, USA), with agitation at 150 rpm. Mammalian Madin-Darby canine kidney (MDCK) cells (NBL-2; ATCC-CCL-34) derived from canine kidney epithelium were obtained from ATCC (Manassas, USA). The cells were cultured in A-DMEM/F12 (1/1, v/v) with 5% fetal calf serum and antibiotics, and maintained in a controlled atmosphere at 37 °C and 5% CO_2_.

#### Proteins

The OlyA6, OlyA6-mCherry, and Δ48PlyB (henceforth PlyB) recombinant proteins were produced as described previously^30,31^. The EryA, EryA-mCherry, PlyA2, and Cry34Ab1 recombinant proteins were produced following the protocols for OlyA6 and OlyA6-mCherry, with slight modifications, as follows. *Escherichia coli* BL21(DE3) cells containing the respective plasmids were grown in Terrific Broth (1.2% tryptone, 2.4% yeast extract, 0.4% glycerol), and protein expression was induced using 0.5 mM isopropyl 1-thio-β-D-galactopyranoside, overnight at 20 °C. The supernatant of the lysed bacterial cells was centrifuged for 30 min at 100 000 × *g* and 4 °C, and incubated with Ni^2+^-NTA beads. Cry34Ab1 was further purified using gel filtration, on Superdex 75 (GE Healthcare, UK) in 0.2 M Na-citrate, pH 4.0. The *Pleurotus* proteins were dialyzed against 20 mM Tris-HCl, pH 8.0, overnight at 4 °C, and further purified on a MonoQ anion exchange column (GE Healthcare, UK). The proteins were eluted with a 0 to 250 mM NaCl gradient in the same buffer. Protein concentrations were determined at 280 nm using a microvolume spectrophotometer (Nanodrop2000; Thermo Scientific, USA). Protein size and purity were determined using sodium dodecyl sulphate polyacrylamide gel electrophoresis (SDS-PAGE; Bio-Rad, USA) on homogeneous 12% acrylamide gels. The protein was then stained with SimplyBlue SafeStain (Thermo Fisher Scientific, USA), or detected with anti-His antibodies after Western blotting (Qiagen, Germany).

#### Target insects

Spotted wing fruit flies (*Drosophila suzukii* [Matsumura]) were laboratory reared from a colony initially provided by Julius Kühn (Institute for Biological Control, Darmstadt, Germany). They were reared in 30 × 30 × 30 cm plastic insectaria in a growth chamber at 22 ± 1 °C and 77% relative humidity, and a photoperiod of 14 h/10 h (light/dark). The *Drosophila* were provided with tap water and solid artificial food medium (20 g agar, 20 g sucrose, 10 g wheat flour, 50 g dry baker’s yeast, 500 mL tap water, 400 g grated organic apples, 500 mL organic apple juice, 50 mL apple vinegar, and 4 g nipagin (methyl 4-hydroxybenzoate; Sigma–Aldrich).

Western corn rootworm (*Diabrotica virgifera virgifera* LeConte) beetles were collected from corn fields from central Slovenia a day prior to the experiments. WCR larvae from a US nondiapausing colony were reared in the insectary of the Agricultural Institute of Slovenia. The initial eggs were obtained from CABI Europe (Switzerland) at the Plant Protection Directorate, Hungary.

Individuals of the grain aphid *Sitobion avenae* Fabricius were collected from winter wheat (*Triticum aestivum* L.) fields in central Slovenia. The *S. avenae* were reared on wheat seedlings in a growth chamber at 22 ± 1 °C and 77% relative humidity, and a photoperiod of 14 h/10 h (light/dark). These aphids have been kept in culture for more than 1 year.

The Colorado potato beetle (*Leptinotarsa decemlineata* Say; CPB) larvae were collected from potato fields a day prior to the experiments, and divided into two groups: young larvae (L1 + L2) and old larvae (L3 + L4). The pronotum was entirely black for L1 + L2. For L3, the anterior margin of the pronotum appeared orange-brown, while for L4, about half of the pronotum was light brown, anteriorly.

Mealworms (*Tenebrio molitor* L.) and greater wax moths (*Galleria melonella* L.) were mass reared in a growth chamber at 30 ± 1 °C and 55% relative humidity, and in the dark, in the insectary of the Agricultural Institute of Slovenia.

### Methods

#### Protein sedimentation assay with multilamellar lipid vesicles

Multilamellar vesicles were prepared with different lipid molar ratios (final concentration, 5 mg/mL) in 140 mM NaCl, 20 mM Tris, 5 μM EDTA, pH 8.0, as described previously^60^. The aegerolysins were added to these vesicles at a 1/10 (w/w) ratio, and incubated for 30 min on a rotary shaker (600 rpm/min) at 25 °C, followed by centrifugation for 60 min at 60 000 × *g* and 4 °C. The supernatants contained the unbound aegerolysins, and they were transferred to vials, and precipitated with 20% trichloroacetic acid. After a 10-min incubation on ice, the precipitated proteins were sedimented by centrifugation at 14 300 × *g* for 12 min at 4 °C, and the pellets were washed twice with 300 μL ice-cold acetone. The fractions containing the free and bound aegerolysin proteins were diluted with an equal volume of nonreducing SDS sample buffer (20 mM Tris-HCl, pH 8.0, 5% [w/v] SDS, 2 mM EDTA, 0.1% [w/v] bromophenol blue), heated to 100 °C for 5 min, and applied to homogeneous 12% acrylamide gels. Proteins were stained with SimplyBlue SafeStain (Thermo Fisher Scientific, USA).

The lipid molar ratios and total lipid concentrations in lipid vesicle suspensions were determined colorimetrically using free cholesterol E and phospholipids C kits (Wako Pure Chemical Industries, Japan), and CPE concentrations were determined by quantification of the amine group^65^.

#### Surface plasmon resonance-based binding studies

To prepare LUVs, suspensions of multilamellar vesicles were subjected to five freeze–thaw cycles and then extruded through 0.1 µm polycarbonate filters (Millipore, Germany) at 40 °C. The size of the LUVs was estimated using dynamic light scattering (Zetasizer Nano ZSP; Malvern Instruments, Malvern, UK). The monomodal distribution of their hydrodynamic radii ranged from 40 nm to 60 nm.

The aegerolysin/LUV interactions were monitored using a surface plasmon resonance-based refractometer (Biacore X; GE Healthcare, USA) and an L1 sensor chip, with 20 mM Tris, 140 mM NaCl, 5 μM EDTA, pH 7.4, as the running buffer. After the initial cleaning of the chip with regeneration solutions of SDS and octyl β-D-glucopyranoside with 1-min injections at a flow rate of 10 μL/min, the LUVs were bound to the second flow cell of the sensor chip to reach responses of ~7,000 RU. The first flow cell was left empty and was used to control for possible nonspecific binding of the aegerolysin proteins to the chip dextran matrix. Loosely bound LUVs were washed from the surface with a 1-min injection of 100 mM NaOH. The nonspecific binding of the aegerolysin proteins was minimized using a 1-min injection of 0.1 mg/mL bovine serum albumin at a flow rate of 30 μL/min. The appropriate solutions of the aegerolysins and the aegerolysin/PlyB complexes were injected at a flow rate of 10 μL/min for 1 min, and their dissociation was monitored for an additional 6 min. The interactions with LUVs were tested for OlyA6, PlyA2, and EryA at concentrations from 0.25 μM to 5 μM, as well as for the aegerolysin complexes OlyA6/PlyB, PlyA2/PlyB, and EryA/PlyB (aegerolysin/PlyB molar ratio, 12.5/1). Chip regeneration between injections was achieved with 1-min injections of 0.5% SDS and 40 mM β-D-glucopyranoside, with a flow rate of 10 μL/min. Experiments were performed at 25 °C. The data were processed with the BIAevaluation software (GE Healthcare).

#### Binding of fluorescently tagged aegerolysins to cell membranes

The Sf9 and MDCK cells were grown on glass coverslips for 1 day and 2 days, respectively. For the labeling with fluorescently tagged aegerolysins, the cells were first fixed using 2% paraformaldehyde, for 15 min at 25 °C, and then washed with phosphate-buffered saline. Then the cells were incubated with 5 μM OlyA6-mCherry or 5 μM EryA-mCherry for 10 min at 25 °C. In some cases, OlyA6-mCherry was preincubated (10 min at 25 °C) with 5 mM multilamellar vesicles composed of equimolar proportions of SM/cholesterol, CPE/POPC/cholesterol or POPC/cholesterol.

After washing with phosphate-buffered saline, the cells were mounted in Vectashield with 4′,6-diamidino-2-phenylindole (DAPI) for nuclei staining, and observed under a fluorescent microscope (AxioImager Z1) using an oil-immersion objective (63× oil, NA 1.40) and an ApoTome device (Carl Zeiss, Germany), for the generation of optical sections. Images were acquired using the Axio-Vision program (Carl Zeiss, Germany).

#### Permeabilization of the small unilamellar vesicles

Small unilamellar vesicles loaded with calcein at the self-quenching concentration (80 mM) were prepared as described previously^66^. Vesicle permeabilization was determined using a fluorescence microplate reader (Anthos, UK) with excitation and emission set at 485 nm and 535 nm, respectively. Calcein-loaded vesicles composed of various molar proportions of lipids were exposed to different aegerolysins (concentrations range, 0 to 100 μg/mL), alone and combined with PlyB at the molar ratio of 12.5/1. The experiments were run for 1 h at 25 °C. The permeabilization induced by the lytic aegerolysin complexes was expressed as the percentage of the maximal permeabilization obtained by addition of the detergent Triton-X 100 to a final concentration of 1 mM.

#### Cell viability assay

Cell viability assays were performed using the CellTiter-Glo reagent (Promega Corp, USA), following the manufacturer instructions. In brief, Sf9 or MDCK cells were plated in 96-well plates (Costar, USA) at 3.7 × 10^4^ cells/cm^2^ and 3 × 10^4^ cells/cm^2^, respectively, in their respective growth medium. After 48 h, the cells were exposed to the aegerolysins (0.1, 1, 5 μM) alone or in combination with PlyB at an aegerolysin/PlyB molar ratio of 12.5/1, for 30 min. The luminescence was measured using a microplate reader (Safire 2; Tecan, Switzerland). The data from the viability assays are expressed as percentages of the luminescence of treated to untreated cells, as means ± standard error of three independent experiments, each performed in triplicate.

#### Insecticidal tests

The insecticidal tests were carried in accordance with the Slovenian law on animal protection (U.l.RS, 2007). The study was not subject to ethical protocols according to EU legislature (Directive 2010/63/EU).

Western corn rootworm: Western corn rootworm larvae (of a US nondiapausing strain) were provided with the southern corn rootworm artificial diet (Frontier Agricultural Sciences, USA; product F9800B; 13.8 g), with some additions. These included agar (1.5 g), lyophilised corn roots (0.63 g), methylene blue solution (product 50484; 100 µL; Sigma–Aldrich, Germany), and demineralized water (88 mL). The pH of the diet was adjusted to 8.0 using 10 M NaOH. Two and a half milliliters of the diet was pipetted into individual wells of sterile six-well plates. Once solidified, 100 µL OlyA6/PlyB, PlyA2/PlyB or EryA/PlyB was added, that was spread evenly over the entire surface of the diet in the wells using a sterile glass stirring rod. The plates were then left to dry for 30 min in a laminar flow chamber.

The mean survival time (LT_50_) was determined by applying 100 µL 0.5 mg/mL OlyA6, PlyA2 or EryA, and 0.04 mg/mL PlyB onto the artificial diet, which provided the final concentration of 5.6 µg/cm^2^ of the aegerolysin/PlyB complexes on the diet. Five neonate larvae were placed in each well. The wells were sealed using pressure-activated sealing foil (product 4ti-0560; 4titude, UK), which was perforated five times to allow for gas exchange. Each treatment was performed in two wells of four replicate six-well plates, and the experiment was repeated twice independently, thus using a total of 80 larvae for each treatment. The bioassay was carried out over 7 days, with larva survival recorded on days 1, 3, 5, and 7.

Following the same 7-day procedure, to determine the median lethal dose (LD_50_), six concentrations of the aegerolysin/PlyB complexes were tested, from 0.0 μg/cm^2^ to 11.2 μg/cm^2^. The total sample size tested at each concentration was 15. Additionally, on day 3, the lengths of the living larvae were measured using a motorized, calibrated stereomicroscope (M205 C; Leica, Germany). The bioassays were performed in a growth chamber at 22 ± 1 °C and 77% relative humidity, and with a photoperiod of 14 h/10 h (light/dark). Buffer was used as the negative control (20 mM Tris, 0.5% glycerol, pH 8.0), with the positive control of 0.5% dilution of the insecticide Decis (active ingredient, 2.8% [w/w] deltamethrin; Bayer CropScience, Germany).

The western corn rootworm beetles collected from corn fields the day prior to the experiments received an artificial diet (pH 7.0) that comprised: sucrose (33 g); alphacel (18.6 g); casein (15.9 g); soy flour (12.4 g); wheat germ (12.4 g); brewer’s yeast (5 g); vitamin mix (Vanderzant, 1.2 g; MP Biomedicals, USA); salt mix (Wesson, 1.2 g; Careforde Inc, USA); cholesterol (0.3 g); glycerol (11 mL); and demineralized water (82.5 mL). The recombinant aegerolysin proteins for the bioassays were mixed with this diet (1/1, v/v), to a final concentration of 0.5 mg/mL aegerolysin and 0.04 mg/mL PlyB. Thirty microliters of this mixture was pipetted into each well of a six-well plate. A single WCR adult was placed into each well. Three replicate six-well plates were performed per treatment, and the experiment was repeated twice independently, for a total of 36 beetles for each treatment. The bioassay was performed in a growth chamber at 22 ± 1 °C and 77% relative humidity, and a photoperiod of 14 h/10 h (light/dark). The buffer (20 mM Tris, 0.5% glycerol, pH 8.0) mixed with the artificial food (1/1, v/v) was used as the negative control, and a 0.5% dilution of the insecticide (Decis) was mixed with the artificial food (1/1, v/v) as the positive control. The bioassay was carried out over 7 days, with the survival recorded daily. Additionally, the individual aegerolysins and PlyB were tested for their potential toxicity for the WCR larvae and beetles.

Colorado potato beetle: The Colorado potato beetle larvae collected from potato fields the day prior to the experiments were divided into two groups based on their pronotum coloring: young larvae (L1 + L2), and old larvae (L3 + L4). The larvae fed on 14-mm-diameter potato leaf discs that were treated by soaking in OlyA6/PlyB, PlyA2/PlyB and EryA/PlyB (0.5 mg/mL aegerolysin, 0.04 mg/mL PlyB) for 5 min. The calculated concentration of aegerolysin/PlyB on the leaf surface was 9.0 µg/cm^2^. A single treated leaf disc and a CPB larva were placed in each well of a six-well plate. Every second day, the relevant freshly treated potato leaf discs were provided. Two replicate six-well plates per treatment were performed, and the experiment was repeated three times independently, giving a total of 36 larvae of each group for each treatment. The bioassay was performed in an incubator at 22 ± 1 °C and 77% relative humidity, and a photoperiod of 14 h/10 h light/day. The buffer-treated (20 mM Tris, 0.5% glycerol, pH 8.0) leaf discs were used as the negative control, and treatment with a 0.1% dilution of insecticide (Actara 25 WG; active ingredient, 25% [w/w] thiametoxam; Syngenta, Switzerland) was the positive control. The survival rate was recorded daily over 5 days. The weights of the larvae were recorded on days 1 and 5. Additionally, the individual aegerolysins, PlyB, and buffer were tested for potential toxicity against the CPB larvae. The insecticidal tests for the other insects tested were carried out as described in Supplementary File S1.

Insecticidal data analysis: The time-based larvae and beetle mortality were analyzed using Kaplan-Meier survival analysis and nonlinear regression. When multiple survival curves were compared, the significance threshold was corrected according to the Bonferroni method. The effects of the aegerolysin proteins on the WCR larva lengths and the CPB larva weight changes were analyzed using Kruskal-Wallis tests, followed by Dunn’s *post-hoc* tests. The data were analysed using GraphPad Prism (GraphPad Software, Inc., USA).

## Supplementary information

Supplementary material

## Data Availability

The datasets generated during and/or analysed during the current study are available from the corresponding author on reasonable request.

## References

[1] Jakka S, Shrestha R, Gassmann A (2016). Broad-spectrum resistance to Bacillus thuringiensis toxins by western corn rootworm (*Diabrotica virgifera virgifera*). Sci. Rep..

[2] Gillette CP (1912). *Diabrotica virgifera* LeConte as a corn rootworm. J. Econ. Entomol..

[3] Kiss, J. *et al*. Monitoring of western corn rootworm (Diabrotica v. virgifera LeConte) in Europe 1992–2003. In: Western corn rootworm: ecology and management (ed. Vidal, S., Kuhlmann, U., Edwards, C. R.) 29–39 (CABI Wallingford, UK, 2005).

[4] Tinsley N, Estes R, Gray M (2012). Validation of a nested error component model to estimate damage caused by corn rootworm larvae. J. Appl. Entomol..

[5] Tinsley N (2015). Estimation of efficacy functions for products used to manage corn rootworm larval injury. J. Appl. Entomol..

[6] Sivčev I, Kljajić P, Kostić M, Sivčev L, Stanković S (2012). Management of western corn rootworm (*Diabrotica virgifera virgifera*). Pestic. Phytomed. (Belgrade).

[7] Kuhlmann U, Burgt WACM (1998). Possibilities for biological control of the western corn rootworm, *Diabrotica virgifera virgifera* LeConte, in central Europe. Biocontrol News Inf..

[8] Toepfer S, Hatala-Zseller I, Ehlers R-U, Peters A, Kuhlmann U (2012). The effect of application techniques on field-scale efficacy: can the use of entomopathogenic nematodes reduce damage by western corn rootworm larvae? *Agr*. Forest Entomol..

[9] Ivezić M (2011). Root compensation of seven maize hybrids due to western corn rootworm (*Diabrotica virgifera virgifera* LeConte) larval injury. *Bulg*. J. Agric. Sci..

[10] Gray ME, Sappington TW, Miller NJ, Moeser J, Bohn MO (2009). Adaptation and invasiveness of western corn rootworm: intensifying research on a worsening pest. Annu. Rev. Entomol..

[11] Adang, M. J., Crickmore, N. & Jurat-Fuentes, J. L. Diversity of Bacillus thuringiensis crystal toxins and mechanism of action. In Advances in insect physiology. (ed. Tarlochan, S. D., Sarjeet S. G.) 39–87 (Academic Press, UK, 2014).

[12] Environmental Protection Agency (EPA). Biopesticides registration action document. Bacillus thuringiensis Cry3Bb1 protein and the genetic material necessary for its production (vector PV-ZMIR13L) in MON 863 corn (OECD Unique Identifier: MON-ØØ863-5). Available online, https://www3.epa.gov/pesticides/chem_search/reg_actions/registration/decision_PC-006484_30-sep-10.pdf (last accessed: 8 May, 2018).

[13] Bravo A, Likitvivatanavong S, Gill SS, Soberón M (2011). *Bacillus thuringiensis*: a story of a successful bioinsecticide. Insect Biochem. Molec..

[14] Gassmann JA (2012). Field-evolved resistance to Bt maize by western corn rootworm: predictions from the laboratory and effects in the field. J. Invertebr. Pathol..

[15] Gassmann JA, Petzold-Maxwell LJ, Keweshan SR, Dunbar WM (2011). Field-evolved resistance to Bt maize by western corn rootworm. Plos ONE..

[16] Chu CC, Sun W, Spencer JL, Pittendrigh BR, Seufferheld MJ (2014). Differential effects of RNAi treatments on field populations of the western corn rootworm. Pestic. Biochem. Phys..

[17] Casagrande RA (1987). The Colorado potato beetle: 125 years of mismanagement. *Am*. Entomol..

[18] Alyokhin A, Baker M, Mota-Sanchez D, Dively G, Grafius E (2008). Colorado potato beetle resistance to insecticides. Am. J. Potato Res..

[19] Huseth AS (2014). Managing Colorado potato beetle insecticide resistance: new tools and strategies for the next decade of pest control in potato. J. Integr. Pest. Manag..

[20] Schellenberger U (2016). A selective insecticidal protein from *Pseudomonas* for controlling corn rootworms. Science.

[21] Wei J-Z (2017). A selective insecticidal protein from *Pseudomonas mosselii* for corn rootworm control. Plant Biotechnol. J..

[22] Sampson K (2017). Discovery of a novel insecticidal protein from *Chromobacterium piscinae*, with activity against western corn rootworm, *Diabrotica virgifera virgifera*. J. Invertebr. Pathol..

[23] Yalpani N (2017). An *Alcaligenes* strain emulates *Bacillus thuringiensis* producing a binary protein that kills corn rootworm through a mechanism similar to Cry34Ab1/Cry35Ab1. Sci. Rep..

[24] Berne S, Lah L, Sepčić K (2009). Aegerolysins: structure, function, and putative biological role. Protein Sci..

[25] Novak M (2014). Fungal aegerolysin-like proteins: distribution, activities, and applications. Appl. Microbiol. Biot..

[26] Butala M (2017). Aegerolysins: lipid-binding proteins with versatile functions. Semin. Cell Dev. Biol..

[27] Tomita T (2004). Pleurotolysin, a novel sphingomyelin-specific two-component cytolysin from the edible mushroom *Pleurotus ostreatus*, assembles into a transmembrane pore complex. J. Biol. Chem..

[28] Sepčić K (2004). Ostreolysin, a pore-forming protein from the oyster mushroom, interacts specifically with membrane cholesterol-rich lipid domains. FEBS Lett..

[29] Resnik N (2011). Desmosome assembly and cell-cell adhesion are membrane raft-dependent processes. J. Biol. Chem..

[30] Ota K (2013). Membrane cholesterol and sphingomyelin, and ostreolysin A are obligatory for pore-formation by a MACPF/CDC-like pore-forming protein, pleurotolysin B. Biochimie.

[31] Skočaj M (2014). Tracking cholesterol/sphingomyelin-rich membrane domains with the ostreolysin A-mCherry Protein. PLoS ONE.

[32] Bhat HB (2013). Binding of a pleurotolysin ortholog from *Pleurotus eryngii* to sphingomyelin and cholesterol-rich membrane domains. J. Lipid Res..

[33] Bhat HB (2015). Evaluation of aegerolysins as novel tools to detect and visualize ceramide phosphoethanolamine, a major sphingolipid in invertebrates. FASEB J..

[34] Crone HD, Bridges RG (1963). The phospholipids of the housefly, *Musca domestica*. Biochem. J..

[35] Itasaka O, Hori T, Uno A, Iwamori M (1973). Occurence of of ceramide phosphorylethanolamine containing hydroxy fatty acid in a bivalve. J. Biochem..

[36] Vacaru AM, van den Dikkenberg J, Ternes P, Holthuis JC (2013). Ceramide phosphoethanolamine biosynthesis in *Drosophila* is mediated by a unique ethanolamine phosphotransferase in the Golgi lumen. J. Biol. Chem..

[37] Kishimoto T, Ishitsuka R, Kobayashi T (2016). Detectors for evaluating the cellular landscape of sphingomyelin- and cholesterol-rich membrane domains. BBA-Mol. Cell. Biol. L..

[38] Lukoyanova N (2015). Conformational changes during pore formation by the perforin-related protein pleurotolysin. PLoS Biol..

[39] Shibata T (2010). Isolation and characterization of a novel two-component hemolysin, erylysin A and B, from an edible mushroom, *Pleurotus eryngii*. Toxicon.

[40] Resnik N (2015). Highly selective anti-cancer activity of cholesterol-interacting agents methyl-β-cyclodextrin and ostreolysin A/pleurotolysin B protein complex on urothelial cancer cells. PLoS ONE.

[41] Qureshi N, Chawla S, Likitvivatanavong S, Lee HL, Gill S (2014). The Cry toxin operon of *Clostridium bifermentans* subsp. *malaysia* is highly toxic to *Aedes* larval mosquitoes. *Appl. Environ*. Microb..

[42] Moellenbeck DJ (2001). Insecticidal proteins from *Bacillus thuringiensis* protect corn from corn rootworms. Nat. Biotechnol..

[43] Kelker M (2014). Structural and biophysical characterization of *Bacillus thuringiensis* insecticidal proteins Cry34Ab1 and Cry35Ab1. PLoS ONE.

[44] Devos Y, Meihls LN, Kiss J, Hibbard BE (2012). Resistance evolution to the first generation of genetically modified *Diabrotica*-active *Bt*-maize events by western corn rootworm: management and monitoring considerations. Transgenic Res..

[45] Narva, K.E., Siegfried, B.D. & Storer, N. Transgenic approaches to western corn rootworm control. In Yellow Biotechnology II (ed. Vilcinskas, A.) 135–162 (Springer, Berlin, 2013). 10.1007/10_2013_195 23604211

[46] Li H (2013). *Bacillus thuringiensis* Cry34Ab1/Cry35Ab1 interactions with western corn rootworm midgut membrane binding sites. PLoS ONE.

[47] Narva KE, Wang NX, Herman R (2017). Safety considerations derived from Cry34Ab1/Cry35Ab1 structure and function. J. Invertebr. Pathol..

[48] Ludwick DC (2017). Minnesota field population of western corn rootworm (Coleoptera: Chrysomelidae) shows incomplete resistance to Cry34Ab1/Cry35Ab1 and Cry3Bb1. J. Appl. Entomol..

[49] Welti R (2007). Lipidomic analysis of *Toxoplasma gondii* reveals unusual polar lipids. Biochemistry.

[50] Guan XL (2013). Biochemical membrane lipidomics during *Drosophila* development. Dev. Cell..

[51] Frankenhuyzen K (2009). Insecticidal activity of *Bacillus thuringiensis* crystal proteins. J. Invertebr. Pathol..

[52] Sakurai N, Kaneko J, Kamio Y, Tomita T (2004). Cloning, expression, and pore-forming properties of mature and precursor forms of pleurotolysin, a sphingomyelin-specific two-component cytolysin from the edible mushroom *Pleurotus ostreatus*. BBA-Gene Struct. Expr..

[53] Yamaji-Hasegawa A, Hullin-Matsuda F, Greimel P, Kobayashi T (2016). Pore-forming toxins: properties, diversity, and uses as tools to image sphingomyelin and ceramide phosphoethanolamine. BBA–Biomembranes.

[54] Rebolj K, Ulrih N, Maček P, Sepčić K (2006). Steroid structural requirements for interaction of ostreolysin, a lipid-raft binding cytolysin, with lipid monolayers and bilayers. BBA–Biomembranes.

[55] Bowling AJ (2017). Histopathological effects of Bt and TcdA insecticidal proteins on the midgut epithelium of western corn rootworm larvae (*Diabrotica virgifera virgifera*). Toxins.

[56] Skočaj M (2016). Characterisation of plasmalemmal shedding of vesicles induced by the cholesterol/sphingomyelin binding protein, ostreolysin A-mCherry. BBA–Biomembranes.

[57] Schlotter, P. & Storer, N. Cry34/35Ab1 mode of action and efficacy. Cost action 862, Bacterial toxins for insect control, WG5 Workshop Salzau, Kiel, Germany, 27th March 2009 (2009).

[58] Felton GW, Workman J, Duffey SS (1992). Avoidance of antinutritive plant defense: role of midgut pH in Colorado potato beetle. J. Chem. Ecol..

[59] Kaiser-Alexnat R (2009). Protease activities in the midgut of western corn rootworm (*Diabrotica virgifera virgifera* LeConte). J. Invertebr. Pathol..

[60] Moreira N, Cardoso C, Dias R, Ferreira C, Terra W (2017). A physiologically oriented transcriptomic analysis of the midgut of *Tenebrio molitor*. J. Insect Physiol..

[61] Berne S (2005). Effect of pH on the pore forming activity and conformational stabillity of ostreolysin, a lipid raft-binding protein from the edible muchroom. Biochemistry.

[62] Terra WR, Ferreira C, Bastos F (1985). Phylogenetic considerations of insect digestion: Disaccharidases and the spatial organization of digestion in the *Tenebrio molitor* larvae. J Insect. Biochemistry.

[63] Pyati P, Bandani AR, Fitches E, Gatehouse JA (2011). Protein digestion in cereal aphids (*Sitobion avenae*) as a target for plant defence by endogenous proteinase inhibitors. J Insect Physiol..

[64] Dimitriadis VK, Pirpasopoulou A (1992). Complex carbohydrate presence in the gut and Malpighian tubules of *Drosophila auraria* larvae (Insecta, Diptera): a cytochemical study. Cytobios..

[65] Barenholz Y (1977). A simple method for the preparation of homogeneous phospholipid vesicles. Biochemistry.

[66] Sepčić K (2003). Interaction of ostreolysin, a cytolytic protein from the edible mushroom *Pleurotus ostreatus*, with lipid membranes and modulation by lysophospholipids. Eur. J. Biochem..

