# Supplementary material for: Pore-forming protein complexes from Pleurotus mushrooms kill western corn rootworm and Colorado potato beetle through targeting membrane ceramide phosphoethanolamine
Source: Sci Rep. 2019 Mar 25;9:5073. doi: 10.1038/s41598-019-41450-4 (PMC6433908; doi:10.1038/s41598-019-41450-4)
Supplement: Supplementary file 1 — Supplementary material [file 41598_2019_41450_MOESM1_ESM.docx]

### Supplemental Information

**Pore-forming protein complexes from *Pleurotus* mushrooms kill western corn rootworm and Colorado potato beetle through targeting membrane ceramide phosphoethanolamine**

***Running title:* *Protein biopesticides from* Pleurotus *mushrooms***

Anastasija Panevska^1^, Vesna Hodnik^1^, Matej Skočaj^1^, Maruša Novak^1^, Špela Modic^2^, Ivana Pavlic^1,3^, Sara Podržaj^1^, Miki Zarić^1^, Nataša Resnik^4^, Peter Maček^1^, Peter Veranič^4^, Jaka Razinger^2,^*, Kristina Sepčić^1,^*

^1^Department of Biology, Biotechnical Faculty, University of Ljubljana, Jamnikarjeva 101, 1000 Ljubljana, Slovenia

^2^Agricultural Institute of Slovenia, Hacquetova 17, 1000 Ljubljana, Slovenia

^3^Department of Biotechnology, University of Rijeka, Radmile Matejčić 2, 51000 Rijeka, Croatia

^4^Institute of Cell Biology, Faculty of Medicine, University of Ljubljana, Vrazov trg 2, 1000 Ljubljana, Slovenia

**

**

**Supplementary Figure S1. Binding of aegerolysins to multilamellar lipid vesicles composed of equimolar lipid mixtures.** The full SDS-PAGE gels from Fig. 1A are shown. The aegerolysins were incubated with multilamellar vesicles and centrifuged as described in the Methods. The supernatant (S) and pellet (P) fractions were subjected to SDS-PAGE and stained with SimplyBlue SafeStain. The size in kilodaltons (KD) is shown. CPE, ceramide phosphoethanolamine; Chol, cholesterol; SM, sphingomyelin; POPC, 1-palmitoyl-2-oleoyl-*sn*-glycero-3-phosphocholine. Arrows denote the monomeric form of aegerolysins (approx. 15 kDa), while higher molecular weight bands correspond to their oligomeric forms^1^.

**

**

**Supplementary Figure S2. Binding of OlyA6 to multilamellar lipid vesicles with varying molar proportions of CPE, POPC, and cholesterol.** The full SDS-PAGE gels from Fig. 1B and Supplementary Fig. S4 are shown. OlyA6 was incubated with multilamellar vesicles and centrifuged as described in the Methods. The supernatant (S) and pellet (P) fractions were subjected to SDS-PAGE and stained with SimplyBlue SafeStain. The size in kilodaltons (KD) is shown. CPE, ceramide phosphoethanolamine; Chol, cholesterol; SM, sphingomyelin; POPC, 1-palmitoyl-2-oleoyl-*sn*-glycero-3-phosphocholine. Arrows denote the monomeric form of aegerolysins (approx. 15 kDa), while higher molecular weight bands correspond to their oligomeric forms^1^.

**

**

**Supplementary Figure S3. Binding of EryA and PlyA2 to multilamellar lipid vesicles with varying molar proportions of CPE, POPC, and cholesterol.** The full SDS-PAGE gels from Fig. 1B and Supplementary Fig. S4 are shown. EryA was incubated with multilamellar vesicles and centrifuged as described in the Methods. The supernatant (S) and pellet (P) fractions were subjected to SDS-PAGE and stained with SimplyBlue SafeStain. The size in kilodaltons (KD) is shown. CPE, ceramide phosphoethanolamine; Chol, cholesterol; SM, sphingomyelin; POPC, 1-palmitoyl-2-oleoyl-*sn*-glycero-3-phosphocholine. Arrows denote the monomeric form of aegerolysins (approx. 15 kDa), while higher molecular weight bands correspond to their oligomeric forms^1^.





**Supplementary Figure S4. Binding specificities of OlyA6 and EryA to multilamellar lipid vesicles with varying molar proportions of CPE, POPC, and cholesterol.** The aegerolysins OlyA6 and EryA were incubated with multilamellar vesicles and centrifuged as described in the Methods. The supernatant (S) and pellet (P) fractions were subjected to SDS-PAGE and stained with SimplyBlue SafeStain. To investigate the effects of the CPE concentration on aegerolysin binding, the vesicles were composed of 0-50 mol% CPE and equimolar ratios of POPC to cholesterol (as indicated). For the effects of cholesterol concentration, the vesicles contained 5 mol% CPE (fixed) and molar ratios of cholesterol to POPC 0 to 47.5 mol% (as indicated). Controls without multilamellar vesicles showed no sedimentation of the aegerolysins. CPE, ceramide phosphoethanolamine; Chol, cholesterol; POPC, 1-palmitoyl-2-oleoyl-*sn*-glycero-3-phosphocholine.


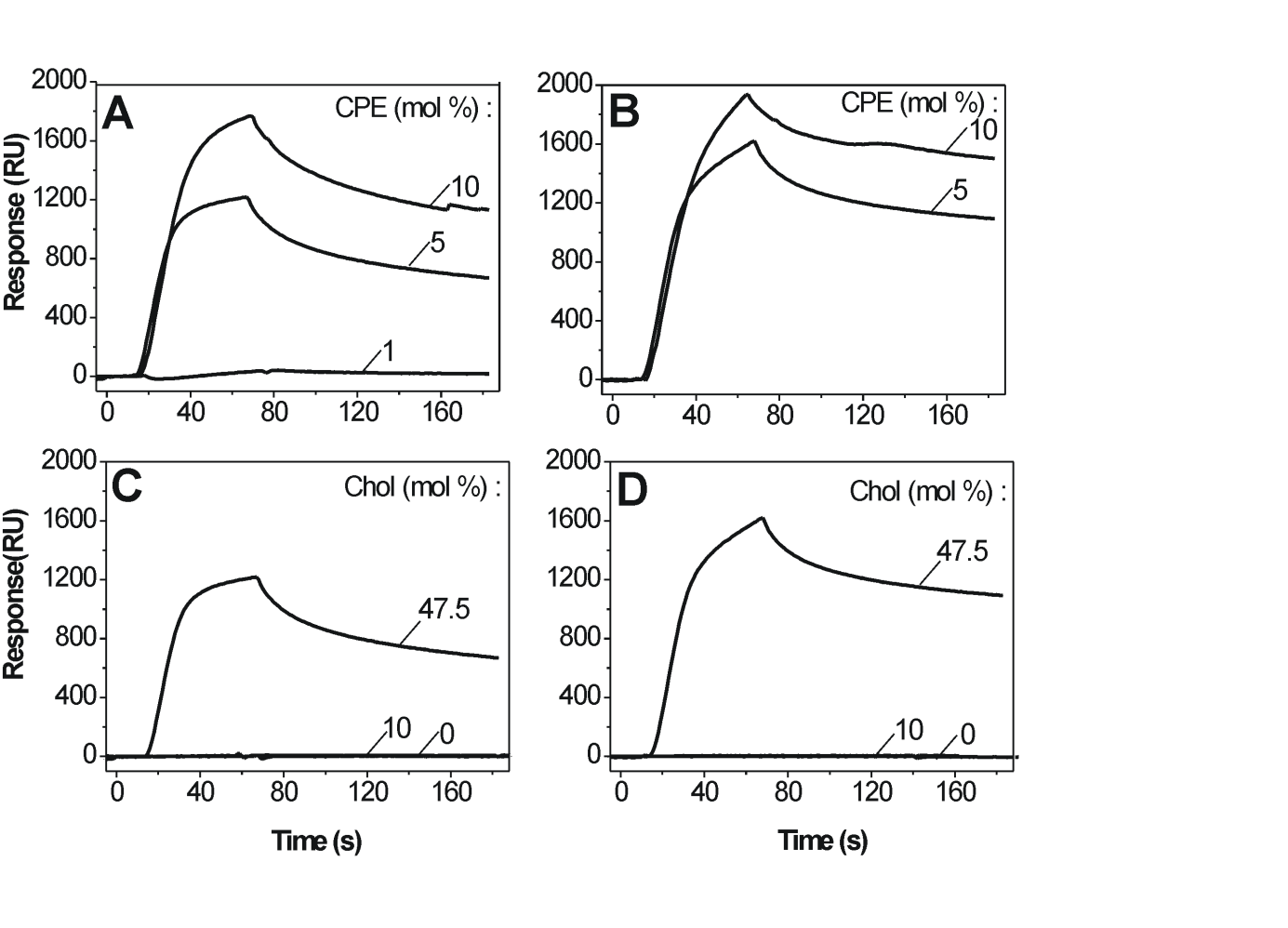


**Supplementary Figure S5. Surface plasmon resonance of the interactions of OlyA6 without and with PlyB with large unilamellar vesicles.** Representative sensorgrams of triplicate analyses for interactions of 1 μM OlyA6 alone (**A, C**) and with PlyB (**B, D**) with vesicles composed of varying molar proportions of CPE (**A**, **B**) and cholesterol (**C**, **D**) (as indicated). Experimental conditions were as described for Figure 2.


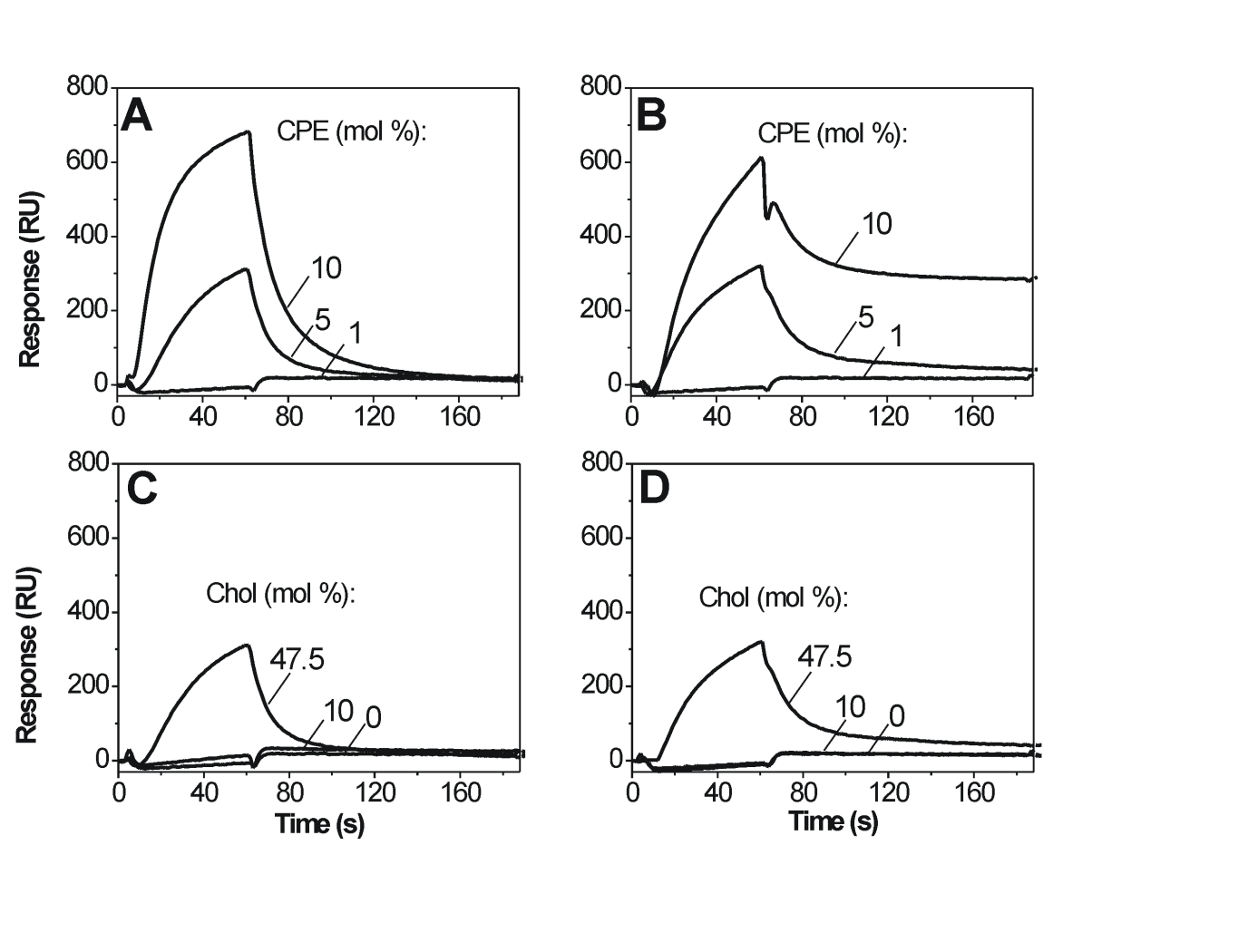


**Supplementary Figure S6. Surface plasmon resonance of the interactions of EryA without and with PlyB with large unilamellar vesicles.** Representative sensorgrams of triplicate analyses for interactions of 1 μM EryA alone (**A, C**) and with PlyB (**B, D**) with vesicles composed of varying molar proportions of CPE (**A**, **B**) and cholesterol (**C**, **D**) (as indicated). Experimental conditions were as described for Figure 2.

**
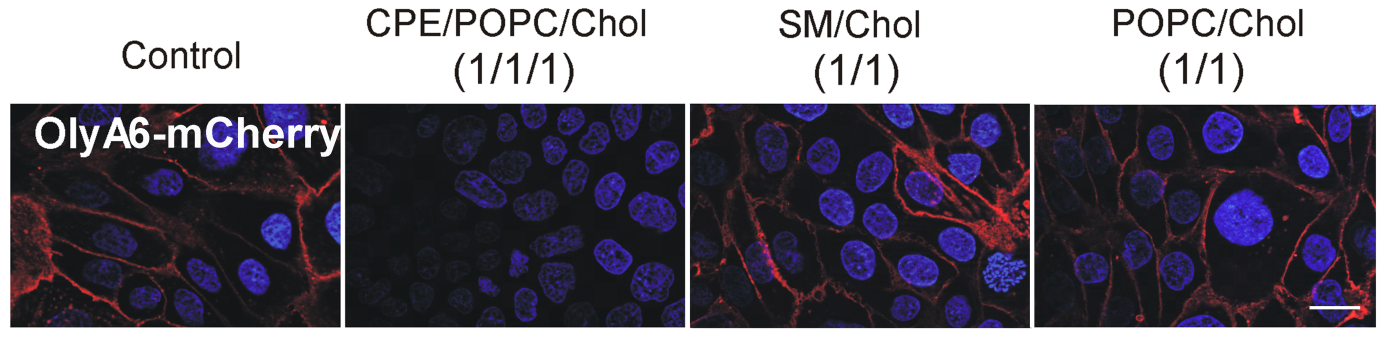
**

**Supplementary Figure S7. Cell surface labelling of MDCK cells with OlyA6-mCherry.** MDCK cells were labelled with OlyA6-mCherry pretreated with SM/cholesterol (1/1), CPE/POPC/cholesterol (1/1/1), or POPC/cholesterol liposomes, as indicated in the Methods section. Scale bar, 20 µm.


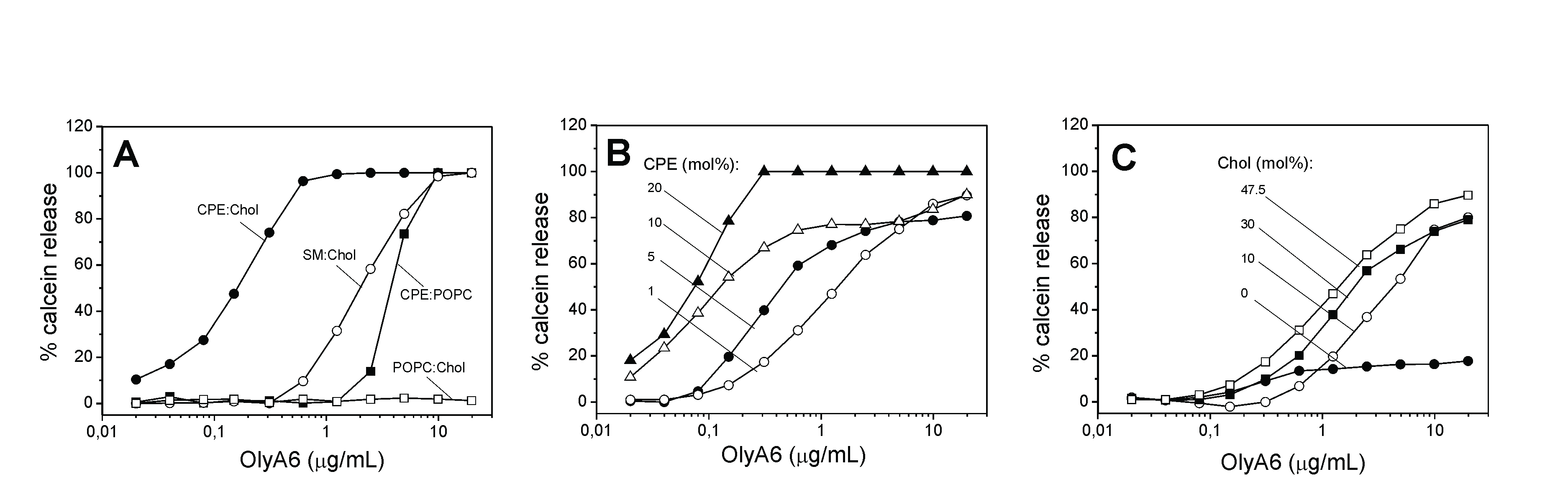


**Supplementary Figure S8**. **Permeabilization of small unilamellar vesicles composed of CPE, POPC, and cholesterol by OlyA6/PlyB.** Fluorescence intensity of calcein released from the lipid vesicles, monitored as described in the Methods. (**A**) Permeabilization of equimolar lipid vesicles composed of sphingomyelin/cholesterol, CPE/cholesterol, CPE/POPC, POPC/cholesterol by OlyA6/PlyB at various concentrations (as indicated). (**B**) Permeabilization of lipid vesicles with varying CPE mol% by OlyA6/PlyB at various concentrations (as indicated). (**C**) Permeabilization of lipid vesicles with varying cholesterol mol% by OlyA6/PlyB at various concentrations (as indicated). (**A**-**C**) OlyA6/PlyB molar ratio, 12.5/1. Experimental conditions were as described for Figure 4. Data are means of three independent measurements (standard error, ≤5%).

#### Supplementary File S1

#### Insecticidal tests

##### ***Methods***

Mealworm (*Tenebrio molitor*) larvae and greater wax moth (*Galleria mellonella*) caterpillars were treated with (bio)insecticides by force-feeding using a microsyringe (Neuros Syringe Gas-tight 1702; Hamilton, USA). Mealworms were force-fed 10 µL and wax moth caterpillars 15 µL OlyA6/PlyB, PlyA2/PlyB and EryA/PlyB (OlyA6, PlyA2, EryA, 0.5 mg/mL; PlyB, 0.04 mg/mL). The same volumes were used for the (bio)insecticides of 1% Actara 25 WG (active ingredient, 25% [w/w] thiametoxam; Syngenta, Switzerland) and 0.05% Lepinox (active ingredient, *B. thuringiensis* var. kurstaki; CBC, Italy) as the positive controls, and the buffer (20 mM Tris, 1% glycerol, pH 7.0) as the negative control.

The mealworm larvae and wax moth caterpillars were obtained from a laboratory rearing program. The bioassays were performed in six-well plates with an individual larva or caterpillar placed in each well. The mealworms fed on oat meal flakes, and the wax moth caterpillars on 10 mg honey/oat/yeast mixture, provided as needed to prevent starvation. Two replicate six-well plates per treatment were performed, and the experiment was repeated three times independently, for a total of 36 larvae and caterpillars for each treatment. The bioassays were performed at room temperature (21.5 ±1.5 °C) and 44%-66% relative humidity, in a closed cardboard box. The survival rates were recorded daily over 24 days.

*Sitobion avenae* aphids were exposed to the aegerolysins/PlyB and chemical insecticide by feeding on an ‘artificial leaf’ system. The artificial leaf was made from the bottom part of a 55-mm-diameter Petri dish covered with two layers of Parafilm (type ‘M’; Bemis, USA). OlyA6/PlyB, PlyA2/PlyB and EryA/PlyB mixtures (OlyA6, PlyA2, EryA, 0.5 mg/mL; PlyB, 0.04 mg/mL) were mixed with aphid artificial diet (1/1, v/v), and were pipetted into the intermembrane space. The artificial diet used was prepared according to Auclair and Cartier^2^, with slight modifications: only the amino acids and sucrose were used. A 1% dilution of the insecticide Actara 25 WG (active ingredient, 25% [w/w] thiametoxam; Syngenta, Switzerland) was used as the positive control, and the buffer (20 mM Tris, 0.25% glycerol, pH 8.0) as the negative control.

The aphids were initially collected from a wheat field, and were subsequently reared on wheat in a growth chamber at 22 ±1 °C and 77% relative humidity, and a photoperiod of 14 h/10 h light/dark). Four N2 nymphs were placed into each Petri dish, which was covered by the first Parafilm layer, onto which a 100 µL drop of the relevant treatment/artificial feed mixture was pipetted; this was then covered by the second Parafilm layer. Five replicate 55-mm-diameter Petri dishes per treatment were performed, and the experiment was repeated twice independently, for a total of 50 aphids per treatment. The bioassays were performed in a growth chamber at 22 ±1 °C and 77% relative humidity, and a photoperiod of 14 h/10 h (light/dark). The survival rates were recorded daily over 3 days.

The *Drosophila suzukii* flies were exposed to the aegerolysins/PlyB and chemical insecticide by mixing 1 g artificial food (20 g agar, 20 g sucrose, 10 g wheat flour, 50 g dry baker’s yeast, 500 mL tap water, 400 g grated organic apples, 500 mL organic apple juice, 50 mL apple vinegar, 4 g nipagin [methyl 4-hydroxybenzoate]; Sigma-Aldrich, Germany; pH 7.0) with 1 mL OlyA6/PlyB, PlyA2/PlyB, and EryA/PlyB ( OlyA6, PlyA2, EryA, 1.0 mg/mL: PlyB, 0.08 mg/mL). A 0.1% dilution of the insecticide Laser 240SC (active ingredient, 23% [w/w] spinosad; Dow AgroScience) was used as the positive control, and the buffer (20 mM Tris, 1% glycerol, pH 7.0) as the negative control.

The flies were reared in a growth chamber at 22 ±1 °C and 77% relative humidity, and a photoperiod of 14 h/10 h (light/dark), as described by Razinger et al.^3^ Fifteen flies (sexual composition, unknown) were transferred into 250 mL plastic food boxes into which 2 g artificial food/treatment solution was added. Three replicate 250 mL plastic boxes per treatment were performed, for a total of 45 flies per treatment. The bioassays were performed in a growth chamber at 22 ±1 °C and 77% relative humidity, and a photoperiod of 14 h/10 h (light/dark). The survival rates were recorded daily over 10 days.

The final numbers of surviving insects were analyzed using ANOVA followed by Dunnett’s multiple comparison post-tests. The data were analysed using GraphPad Prism (GraphPad Software, Inc., La Jolla, CA, USA).

##### ***Results***

The treatments significantly affected the insect survivals in the bioassays: *T. molitor*, F_4, 26_ = 32.0; P <0.0001; *G. mellonella*, F_5, 29_ = 13.4; P <0.0001; *S. avenae*, F_5, 65_ = 11.4; P <0.0001; and *D. suzukii*, F_4, 14_ = 14.0; P <0.0001. However, in all cases, only the insecticides (i.e., Actara, Laser) significantly increased insect mortality (**Fig. S5**).

**References:**

# [1] [Auclair, J.L](https://www.ncbi.nlm.nih.gov/pubmed/?term=Auclair%20JL%5BAuthor%5D&cauthor=true&cauthor_uid=17835604)., and [Cartier, J.J](https://www.ncbi.nlm.nih.gov/pubmed/?term=Cartier%20JJ%5BAuthor%5D&cauthor=true&cauthor_uid=17835604). (1963). Pea aphid: rearing on a chemically defined diet. [Science](https://www.ncbi.nlm.nih.gov/pubmed/17835604) *142*, 1068-1069.

[2] Razinger, J., Fink, K., Kerin, A., Modic, Š., and Urek, G. (2017). Susceptibility of spotted wing drosophila [(*Drosophila suzukii* (Matsumura, 1931)) pupae to entomopathogenic fungi](http://ojs.aas.bf.uni-lj.si/index.php/AAS/article/view/358). Acta Agric. Slov. *109*, 125-134.





Supplementary Figure S9. Survival of *Tenebrio molitor* (A), *Galleria mellonella* (B), *Sitobion avenae* (C), and *Drosophila suzukii* (D) exposed to *Pleurotus* aegerolysins/PlyB and the insecticides Actara (active ingredient, thiamethoxam) and Laser (active ingredient, spinosad), and the bioinsecticide Lepinox (active ingredient, *B. thuringiensis* var. kurstaki). *, P <0.05, between treatments and negative control buffer.

**References:**

# [1] Sepčić, K. *et al.* Interaction of ostreolysin, a cytolytic protein from the edible mushroom *Pleurotus ostreatusI,* with lipid membranes and modulation by lysophospholipids. *Eur. J. Biochem*. 270, 1199-1210 (2003).

# [2] [Auclair, J.L](https://www.ncbi.nlm.nih.gov/pubmed/?term=Auclair%20JL%5BAuthor%5D&cauthor=true&cauthor_uid=17835604). and [Cartier, J.J](https://www.ncbi.nlm.nih.gov/pubmed/?term=Cartier%20JJ%5BAuthor%5D&cauthor=true&cauthor_uid=17835604). Pea aphid: rearing on a chemically defined diet. [*Science*](https://www.ncbi.nlm.nih.gov/pubmed/17835604) 142, 1068-1069 (1963).

[3] Razinger, J. *et al.* Susceptibility of spotted wing drosophila [(*Drosophila suzukii* (Matsumura, 1931)) pupae to entomopathogenic fungi](http://ojs.aas.bf.uni-lj.si/index.php/AAS/article/view/358). Acta Agric. Slov. **109**, 125-134 (2017).
